# Immunotoxicity of β-Diketone Antibiotic Mixtures to Zebrafish (*Danio rerio*) by Transcriptome Analysis

**DOI:** 10.1371/journal.pone.0152530

**Published:** 2016-04-05

**Authors:** Fanghui Li, Hui Wang, Jinfeng Liu, Jiebo Lin, Aibing Zeng, Weiming Ai, Xuedong Wang, Randy A. Dahlgren, Huili Wang

**Affiliations:** 1 School of Life Sciences, Wenzhou Medical University, Wenzhou, China; 2 Key Laboratory of Watershed Sciences and Health of Zhejiang Province, Wenzhou Medical University, Wenzhou, China; University of Liège, BELGIUM

## Abstract

Fluoroquinolones and tetracyclines are known as β-diketone antibiotics (DKAs) because of bearing a diketone group in their molecular structure. DKAs are the most widely used antibiotics to prevent generation of disease in humans and animals and to suppress bacterial growth in aquaculture. In recent years, overuse of DKAs has caused serious environmental risk due to their pseudo-persistence in the environment, even though their half-lives are not long. So far, no reports were concerned with the joint immunotoxicity of DKAs. Herein, we reported on the immunotoxicity of DKAs on zebrafish after a 3-month DKAs exposure using transcriptomic techniques. According to transcriptome sequencing, 10 differentially expressed genes were screened out among the genes related to KEGG pathways with high enrichment. The identified 7 genes showed to be consistent between RNA-seq and qRT-PCR. Due to DKAs exposure, the content or activity for a series of immune-related biomarkers (Complement 3, lysozyme, IgM and AKP) showed the inconsistent changing trends as compared with the control group. Histopathological observations showed that the number of goblet cells increased sharply, the columnar epithelial cells swelled, the nucleus became slender in intestinal villi, and numerous brown metachromatic granules occurred in spleens of DKAs-exposed groups. Overall, both detection of biomarkers and histopathological observation corroborated that chronic DKAs exposure could result in abnormal expression of immune genes and enzymes, and variable levels of damage to immune-related organs. These complex effects of DKAs may lead to zebrafish dysfunction and occurrence of diseases related to the immune system.

## Introduction

β-Diketone antibiotics (DKAs), including fluoroquinolones and tetracyclines, are currently the most widely used antibiotics in humans and animals to prevent generation of disease and to suppress bacterial growth in aquaculture [1–2]. In recent years, overuse of DKAs has caused serious environmental risk due to their pseudo-persistence in the environment, even though their half-lives are not long. In hospital and animal husbandry sewage, DKAs concentrations were as high as 0.2–101.0 mg/L [3]. Such high levels of DKAs in real-world environments pose a severe threat to humans, wildlife and aquatic organisms. DKAs can produce environmental biological impedance, with severe liver, kidney and neurotoxicity effects. Long-term exposure to fluoroquinolones can lead to many adverse effects, such as central nervous system (CNS) toxicity, cardiotoxicity, arthropathy, and so on [4]. In comparison, the long-term use of tetracyclines can result in immune toxicity, feminization, reproductive failure, abortion, etc. For example, tetracyclines lead to fatty liver disease by interfering with the expression of lipid metabolism-related genes and biosynthesis of triglycerides, and reducing β-fatty acid oxidation [5]. A major threat in fluoroquinolone toxicity is the impact of cartilage development in young animals, such as rats, dogs, rabbits and other non-primate animals [6]. Enorxacin could significantly inhibit activities of acetylcholine esterase (AChE), showing its potential neurotoxicity in fish [7]. In detoxification metabolism, the induced production of glutathione (GSH) by DKAs was observed at the end of the hatching period, but the inhibited activities of superoxide dismutase (SOD) occurred in zebrafish (*Danio rerio*) [8].

Previous studies have primarily focused on the toxicity of single DKA species to explore the relevant toxicological mechanisms. However, in real-world aquatic environments, mixtures of several different DKA species may have entirely different toxicological mechanisms, which can lead to complex, combined toxicity actions by DKAs mixtures [9–10]. For *in vivo* experiments, changes in metabolic transformation and distribution of DKAs make the toxicological research more complicated [11–12]. As a result, evaluating the joint toxicity of DKA mixtures is a challenging and practical pursuit necessary to assess toxicity threats to real-world aquatic environments. In this study, we chose three fluoroquinolones and three tetracyclines as representative DKAs species, based on their frequent detection in aquatic environments, to analyze their chronic toxicity to zebrafish (*Danio rerio*) using transcriptomic techniques.

Zebrafish (*Danio rerio*) serve as a preferred toxicity model due to their rapid life cycle, high fecundity, transparent development, and their embryos being amenable to genetic manipulation [13]. Zhang and coworkers [14] found that tetracycline produced oxidative stress and induced apoptosis, which brought about significant developmental delay in zebrafish embryos. Yin and coworkers [9] used proteomic analysis of zebrafish to evaluate long-term trace exposure to DKAs, and found that 47 differential expression proteins compared to the control, and the number of positive proteins was 26 with up-regulation of 14 and down-regulation of 12. Our group previously demonstrated that DKAs were developmentally toxic in the early life stages of zebrafish and impaired individual behaviors [8]. To our knowledge, no previous studies have investigated joint immunotoxicity of DKAs to zebrafish by transcriptomic techniques, therefore providing a new and sensitive approach for assessing immunotoxicological responses.

This study aims to sequence zebrafish in control and mixed DKA-exposed groups to determine differentially expressed genes and to investigate the gene annotation and signaling pathways. Part of the immune-related genes with significantly differential expression were validated by qRT-PCR. Additionally, we studied biomarker and other pathological index changes to probe the combined immunotoxicity effects of joint FQs and TCs-exposure on zebrafish. These results provide a theoretical basis for establishing a systematic toxicological model and evaluating the ecological risks of exposure to mixtures of DKAs in the environments.

## Materials and Methods

### Ethics statement

The use of zebrafish (*Danio rerio*) in this investigation was approved by our university’s IACUC (Institutional Animal Care and Use Committee), and all the experiments were strictly performed on the basis of rules of the IACUC. All dissection was conducted on ice to decrease suffering.

### Chemicals, fish husbandry and exposure protocols

Six kinds of DKAs were selected as the exposed chemicals: ofloxacin, ciprofloxacin, enrofloxacin, doxycycline, chlortetracycline and oxytetracycline with purity of 99% except for 95% for chlortetracycline. They were gratis supplied by Amresco (Solon, OH, USA), and their structures are shown in S1 Fig. Wild-type (AB strain) zebrafish (*Danio rerio*) were raised in conditioned water, which were dechlorinated and filtered, at 28°C with a 14:10-h light/dark cycle (lights on at 8 am) [15]. Water was dechlorinated and filtered by reverse osmosis (pH 6.5–7.5), and Instant Ocean band of salt was added to raise the specific conductivity to 450–1000 μS/cm. Zebrafish were fed twice daily with live *Artemia* (Jiahong Feed Co., Tianjin, China) and dry flake diet (Zeigler, Aquatic Habitats, Apopka FL, USA) [10]. The fertilized and normal embryos were inspected and staged for subsequent experiments under a stereomicroscope according to Kimmel and coworker’s method [16]. A series of DKAs levels (0, 6.25, 12.5, 25 and 50 mg/L with equal weight concentrations and volumes for each of the six DKA species) were selected as the exposure concentrations. At 6 dpf (days post fertilization), well-hatched zebrafish were transferred to 2 L tanks until 30 dpf. For each day, the renewed DKAs solution was added to keep constant DKAs-exposure level (S2 Fig) [9]. The control (with survival rate of >95%) and treatment groups were performed in triplicate. At 30 dpf, each group including control and treatment was respectively transferred from 2L to 12 L tank. The DKA-exposure concentration and the renewing frequency of DKAs in 12L tank remained unchanged as those in 2L tank.

### Preparation of biological samples

Biochemical activity was determined in isolated tissues from adult zebrafish at 90 dpf. After DKAs exposure, zebrafish in three biological replicates were anesthetized with 0.03% tricaine (buffered MS-222). Each biological replicate included 9 zebrafish, and thus 27 zebrafish were used for RNA-seq, qRT-PCR, each biomarker analysis and pathological observation. Heparin was used to prevent clotting of the blood in the course of collection of peripheral blood from caudal vein of each fish. Concurrently, the gills, small intestines and spleens were dissected over ice.

### Total RNA extraction and building of DNA library

Zebrafish in control and treatment (6.25 and 12.5 mg/L) groups were randomly selected after DKAs exposure at 90 dpf, and rinsed with phosphorous buffer solution. We performed simple anatomy of the whole fish, i.e., removing gill, fin and tail tissue, while retaining visceral mass, head, spine and other nerve systems. The retained tissues were thoroughly ground in liquid nitrogen, and centrifuged at 4000 *g*, 4°C for 10 min to extract total RNA, using Trizol (Invitrogen, Carlsbad, CA, USA) according to the manufacturer's procedures. The quantity and purity of total RNA were analyzed by employing a Bioanalyzer 2100 and RNA 6000 Nano LabChipKit (Agilent, Santa Clara, CA, USA) with RIN number >7.0. Poly (A) mRNA was isolated by poly-T oligo attached magnetic beads (Invitrogen). The purified mRNA was fragmented into small pieces by divalent cations under elevated temperature. Then the final cDNA library was produced by the reverse-transcription of the cleaved RNA fragments on the basis of the mRNA-Seq sample preparation kit (Illumina, San Diego, USA), and the average 300±50 bp insert size was used for the paired-end libraries. Finally, the paired-end sequencing was performed on an Illumina Hiseq2000/2500.

Prior to assembly, the low quality reads (1, reads containing sequencing adaptors; 2, reads containing sequencing primer; 3, nucleotide with q-scores lower than 20) were removed. Next, the cleaned and paired-end reads were produced.

### Functional annotation and analysis of differentially expressed genes

We handled the aligned read files by Cufflinks (http://cole-trapnell-lab.github.io/cufflinks/), which measured the relative abundance of transcripts by taking advantage of the normalized RNA-seq fragment counts. The unit of measurement was Fragments Per Kilobase of exon per Million fragments mapped (FPKM). Sequenced sample genes were analyzed at different expressions based on the FPKM of valid reads. In this analysis, the threshold for differentially expressed genes was set at a *p*-value≤0.05. We defined genes with *p*-value≤0.05 and ≥2-fold differential expression between a pair of samples at a false discovery rate (FDR) of 5% as significantly differential expression genes. Cluster analyses of differentially expressed genes were used to determine the clustering model of gene regulation under different experimental conditions. According to a similar degree of gene expression profile, cluster analyses can visually demonstrate representation of gene expression in different samples, thereby obtaining biologically relevant information (MEV4-9 software, http://www.tm4.org/mev.html). Based on genome (Zv9), we used Kyoto Encyclopedia of Genes and Genomes (KEGG) pathway analysis to classify and annotate the metabolic pathways of differentially expressed genes (http://www.genom.jp/kegg). Gene function for COG (Cluster of Orthologous Groups of proteins) or KOG (Eukaryotic Orthologous Groups of proteins) classification provide an intuitive understanding of the related functions for the significant differentially expressed genes. As a result, COG or KOG classification can provide guidance for interpretation of biological experiments.

Interactions and coordination exist among genes in organisms, which exert their biological functions and maintain physiological activities. Pathway-based analysis of genes provides a better understanding of gene biological functions. Using the hypergeometric test and comparing with the background of the entire genome in KEGG.

KEGG analysis (p-value ≤0.01) was obtained from the KEGG automatic annotation web service (http://www.genom.jp/kegg), which is a powerful tool for analyzing metabolic pathways *in vivo*. This allowed us to search for all metabolic pathways, as well as determine the annotation of the catalytic reaction enzymes in full steps.

### Real-time PCR analysis for differentially expressed immune-related genes

qRT-PCR was used to verify the differentially expressed genes of zebrafish at 90 dpf. The treated protocol of whole fish was similar as that of RNA-seq. Trizol reagent was used to extract total RNA and the measured results of total RNA by the Nanodrop should meet the requirements: OD_260_/OD_280_ >1.8 and OD_260_/OD_230_ >1.5. Primer sets were designed using Primer Premier 5.0 software and were synthesized by Shanghai Sangon Biotechnology Company (Shanghai, China) (S1 Table). Then, cDNA was prepared from total RNA using a Prime-Script RT reagent Kit (TaKaRA, Dalian, China) according to the manufacturer’s instructions. RT-PCR reaction conditions were 37°C for 15 min, 85°C for 5 s; and 4°C cooling. The real-time quantitative PCR kit in this experiment was All-in-One^**™**^ qPCR Mix of GeneCopoeia^**™,**^ All-in-One^**™**^ qPCR Mix used SYBR green, and qRT-PCR was performed using Bio-Rad instrument (Bio-Rad CFX96, Hercules, USA). qRT-PCR procedures were as follows: initial denaturation, 95°C for 10 min; 95°C denaturation for 10 sec, 60°C annealing for 20 sec, 72°C extension for 15 sec, 45 times circulation of this step; melting curve reading: 72°C~95°C, heating rate 0.5°C/ 10 sec; 25°Ccooling for 30 sec. One single peak of all the melting curves in the tested range showed good specificity. The negative controls showed no detectable fluorescence signal. For calculating expression levels, normalization was performed by subtracting the mean threshold cycle (Ct) values for the β-actin gene, which served as internal control, from the mean Ct value of the target gene (ΔCt value). The ΔCt values of treated samples were calibrated against the untreated (control) ΔCt values for all target genes. The relative amount of target molecules relative to the control was calculated by 2^-ΔΔCt^. The mRNA expression level of the different target genes are expressed as fold change according to the formula: 2^- (ΔCt(treated sample)—ΔCt(untreated sample))^.

### Detection of biomarkers and histopathological observation

Changes in immune-related enzymes were detected including alkaline phosphatase (AKP) in blood, lysozyme activity, and concentrations of IgM and Complement 3 in this study. Lysozyme and AKP activity, C3 and IgM concentration were determined using test kits following manufacturer’s specification (Nanjing Jiancheng Bioengineering Institute, Nanjing, China).

In hispathological analyses, we selected two methods, paraffin sections and electron microscopic observations, paraffin section: isolated tissues were fixed in 4% paraformaldehyde (PFA) overnight. Tissues were subsequently paraffin embedded. These paraffin blocks were then microtome sectioned into 5 μM thin sections onto microscope slides. After drying sections were stained with haematoxylin and eosin (H&E) staining was performed using a standard protocol. Electron microscopic observation of tissue damage was examined using transmission electron microscopy (TEM 10; Zeiss, Jena, Germany). The spleen and gill were dissected in control and treatment groups, cut into 1 mm^3^ tissue blocks and fixed in glutaraldehyde at 4°C. A series of pretreatment procedures, such as poaching, fixing, dehydrating, embedding and sectioning, were performed to prepare tissues for TEM observation.

### Statistical analysis

During the whole experimental period, three biological and three technological replicates were maintained for the sake of accuracy and reproducibility, and each biological replicate included 9 zebrafish. As a result, 27 zebrafish (3×9) were used for qRT-PCR, each biomarker analysis and hispathological observation. Real-time quantitative data were treated by 2^**-ΔΔCT**^. One-way analysis of variance (ANOVA) was used to detect DKAs treatment effects, followed by pairwise Dunnett’s tests or Tukey's tests to independently compare the different DKAs levels to the control group.

## Results

### RNA sequencing quality

RNA sequencing met the quality requirements for all control and treatment groups: OD_260_/OD_280_ >1.8, OD_260_/OD_230_ >1.5 and RIN number >7.0. We performed paired-end sequencing of control and two DKAs treatment groups on an Illumina Hiseq2000/2500 to obtain transcriptome data for the three groups. This produced 27.62 gigabases (Gb) of valid reads, demonstrating that the sequencing data volume for each group was larger than 8 Gb (S2 Table). Each sample’s sequencing Q20 was higher than 99%, indicating good sequencing quality. The deep sequencing produced 71,532,662 (control), 65,426,852 (6.25 mg/L treatment) and 84,020,924 (12.5 mg/L treatment) valid reads. All sequencing reads were mapped to the reference genome for zebrafish Zv9 (ftp://ftp.ensembl.org/pub/release-66/fasta/danio_rerio/dna/). Using Tophat2, the reads mapped to the reference genome were 27,752,966 (38.8%), 36,032,694 (55.1%) and 45,610,683 (54.3%) in control, 6.25 mg/L and 12.5 mg/L treatments, respectively. According to region information in the reference genome, the mapped regions of valid data were defined as exon, intron and intergenic regions. In normal circumstances, the maximum percentage of sequences should be in the exon region. This was the case for our investigation as the percentage of reads mapped to the exon region of the genome was more than 90%. This demonstrates that subsequent analyses for gene expression levels can be reasonably performed. As a result, we used FPKM (Fragments Per Kilobase of exon model per Million mapped reads) to conduct a statistical analysis for the known gene expression abundance in the different samples (S2 Table).

### Functional analyses of differentially expressed genes

**Fig 1:**
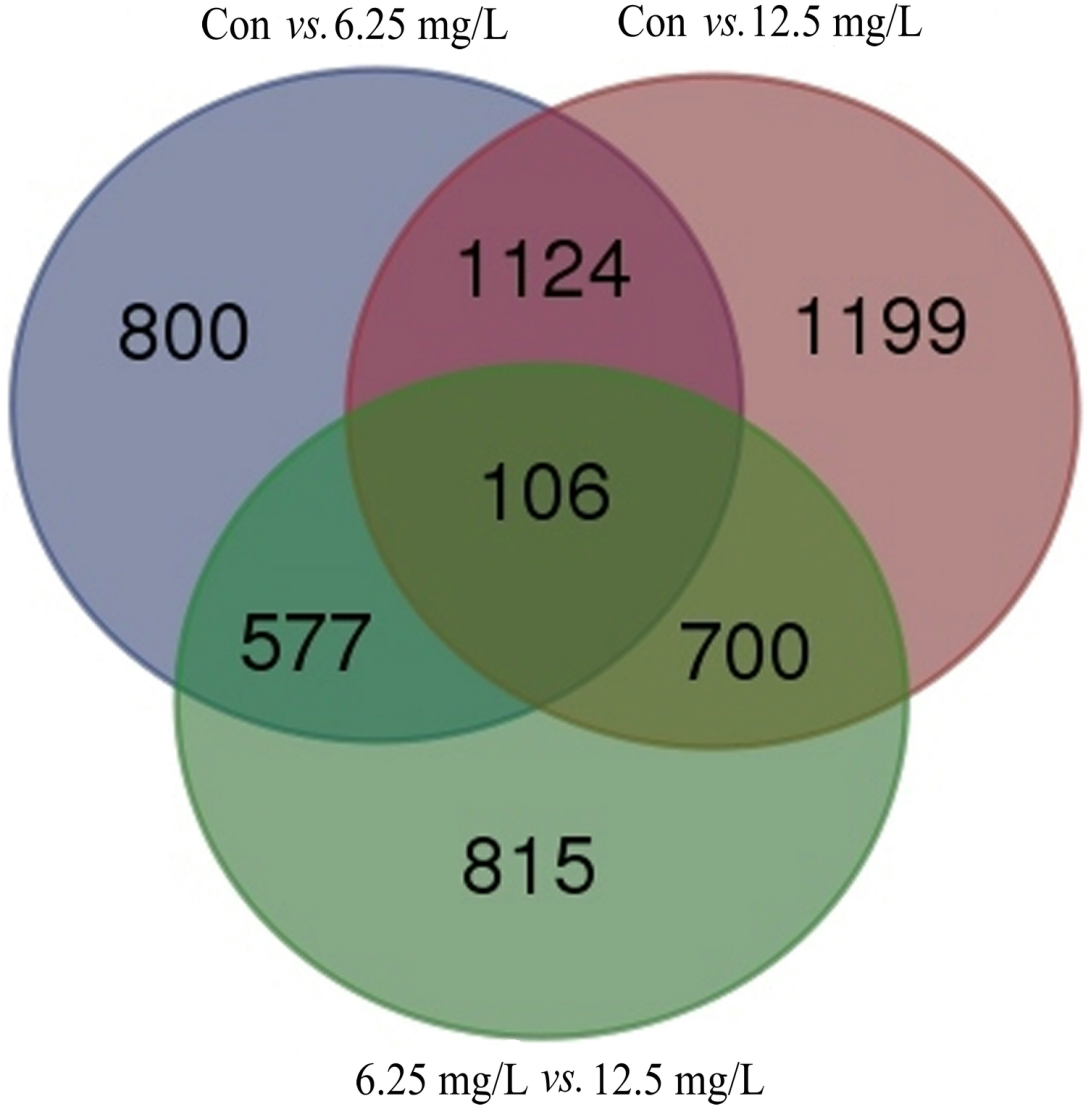
Venn diagram of the differentially expressed genes between the different comparisons. Note: (1) The sum of the number in each circle represents the amount of differentially expressed genes between the different comparisons (control *vs*. 6.25 mg/L; control *vs*. 12.5 mg/L and 6.25 *vs*. 12.5 mg/L); (2) The overlapping number stands for the mutual differentially expressed genes between the different comparisons (control *vs*. 6.25 mg/L; control *vs*. 12.5 mg/L and 6.25 *vs*. 12.5 mg/L).

For the three mutual comparison groups (control *versus* 6.25 mg/L treatment; control *versus* 12.5 mg/L treatment; 6.25 mg/L *versus* 12.5 mg/L treatments), there were 106 genes with significant co-differential expression (*p*<0.05, fold-change≥2) in comparison of any two groups (Fig 1 and S3 Table). For the comparison group of control *versus* 6.25 mg/L treatment, 2607 differentially expressed genes were found, among which 1886 genes were up-regulated and 721 ones were down-regulated. Similarly, there were 3129 differentially expressed genes when compared control with 12.5 mg/L treatment, among which 2221 genes were up-regulated and 908 ones were down-regulated. However for the 106 co-differentially expressed genes in the three comparison groups, the up and down-regulated genes were 58 and 48 in 6.25 mg/L treatment group, while those were 56 and 50 in 12.5 mg/L treatment group, respectively, as shown in S3 Fig.

**Fig 2:**
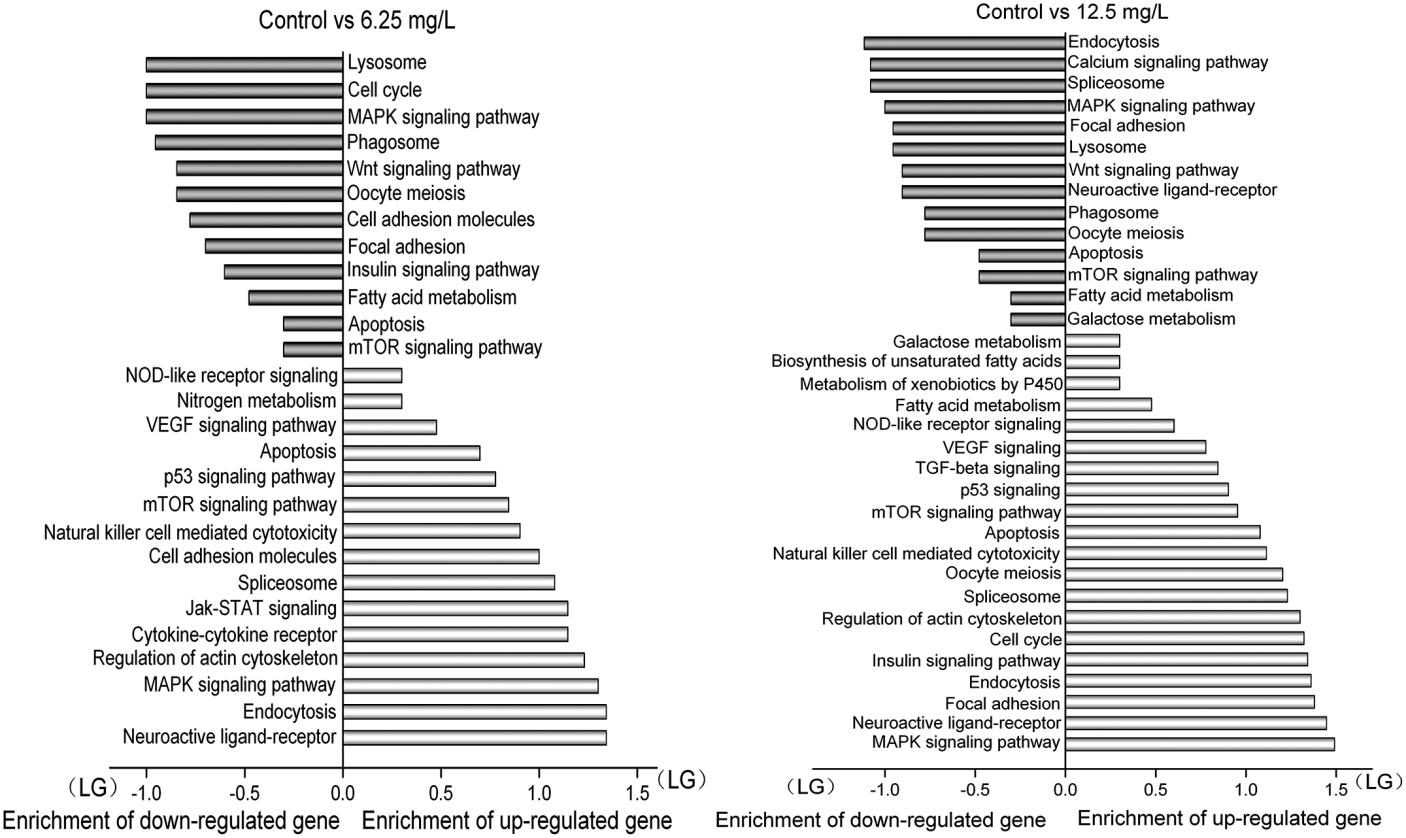
KEGG pathway enrichment analysis of the differentially expressed genes in the 6.25 mg/L and 12.5 mg/L treatment when compared with the control group. Note: (1) The enrichment value is based on lg^(gene-enriched number)^; (2) The positive value in horizontal axis denotes the up-regulated genes; (3) The negative value in horizontal axis denotes the down-regulated genes.

The noted KEGG pathway enrichment analysis was conducted on the basis of the number of differentially expressed genes. The up-regulated genes in the 6.25 mg/L treatment mainly participated in neuroactive ligand-receptor interaction, endocytosis, MAPK signaling pathway and regulation of actin cytoskeleton. In comparison, the down-regulated genes in the 6.25 mg/L treatment primarily took part in lysosome, cell cycle and MAPK signaling pathway (*p*≤0.01, Fig 2). For the 12.5 mg/L treatment, the up-regulated genes mainly participated in MAPK signaling pathway, neuroactive ligand-receptor interaction and focal adhesion pathway, while the down-regulated genes primarily took part in endocytosis, calcium signaling pathway and spliceosome when compared with the control group.

Among the 106 co-differentially expressed genes, 34 KEGG pathways linked and 25 pathways (*p*≤0.01) were selected as the most significantly differential pathways (S4 Table). Focal adhesion, MAPK signaling, regulation of the actin cytoskeleton, toll-like receptor signaling, VEGF signaling, natural killer cell mediated cytotoxicity, apoptosis, ErbB signaling and ubiquitin mediated proteolysis were all involved in regulation of the immune system.

**Fig 3:**
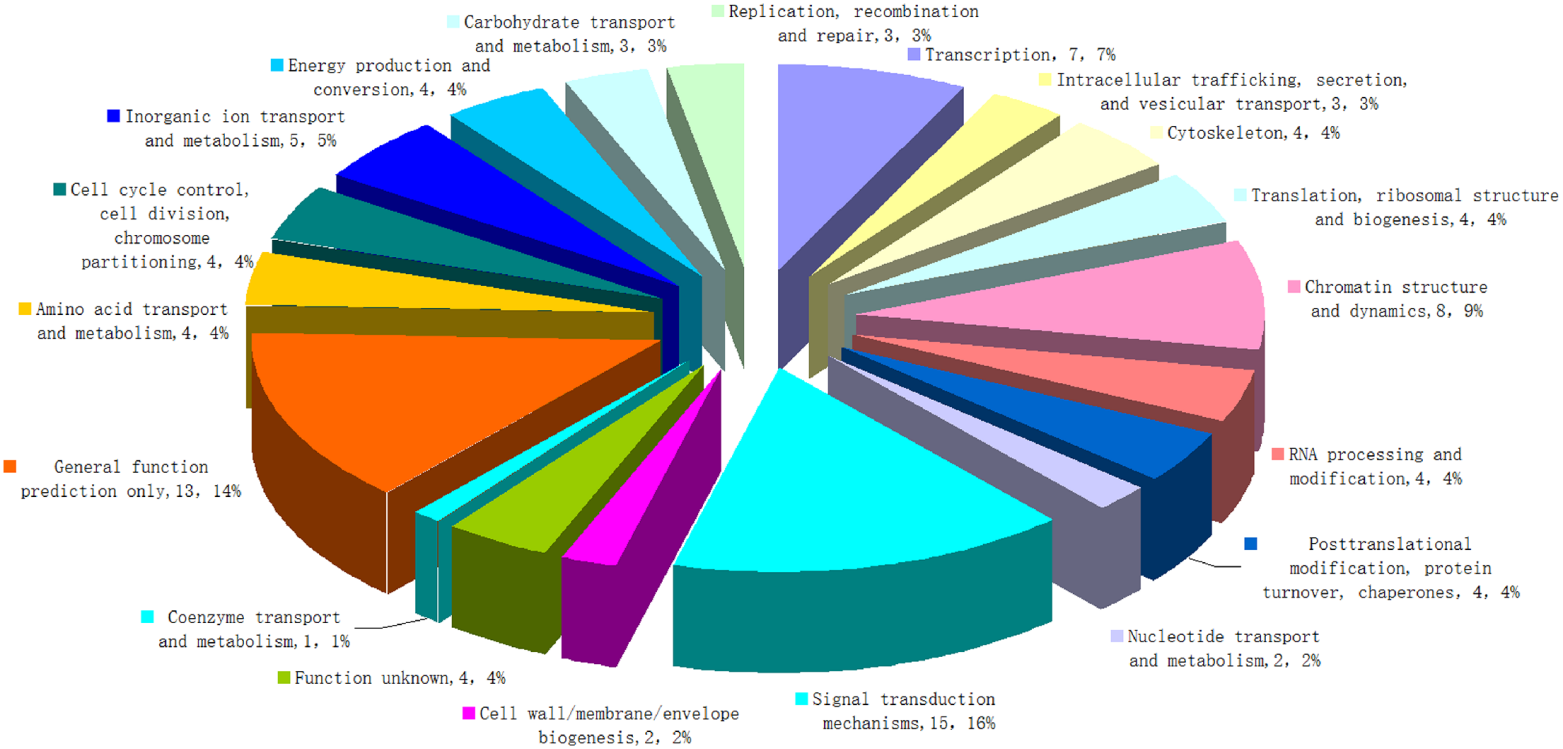
KOG analyses of the differentially expressed genes.

Information on the ortholog of gene products and functional classification for the differentially expressed genes was determined using KOG online software (). Among the 106 co-differentially expressed genes, 94 genes, accounting for 89%, were annotated in KOG. The proteins encoded by the differentially expressed genes were mainly classified as signal transduction mechanisms followed by chromatin structure and dynamics, and transcription regulation (Fig 3). Cluster analysis of the 106 co-differentially expressed genes provided important information for understanding differential expression between treatment and control groups.

### Screening of differentially expressed genes and qRT-PCR analysis

**Fig 4:**
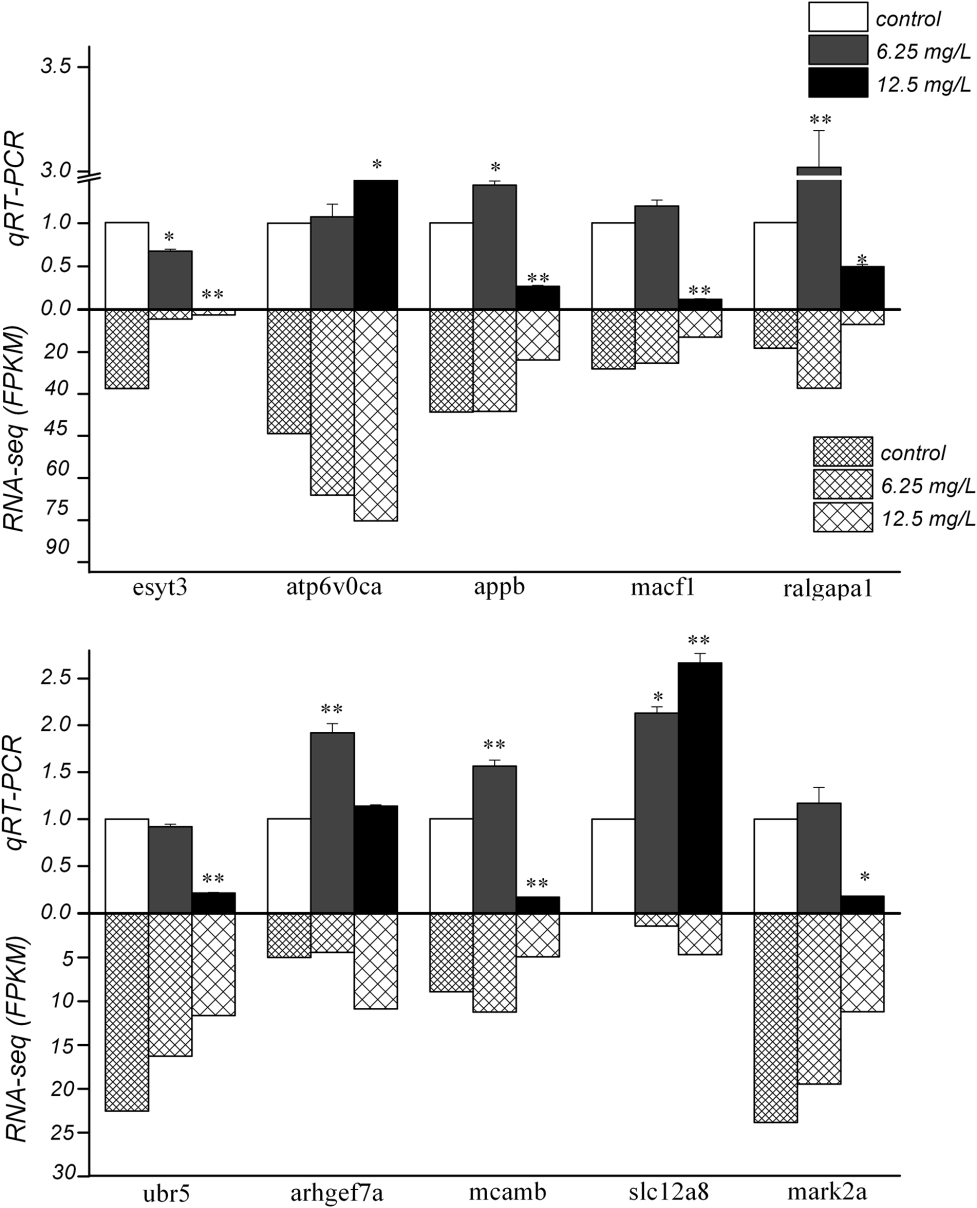
The differential expression of the 10 selected genes for the control and DKAs-expousure treatment group by qRT-PCR. Note: (1) * indicates level of significance at *p*<0.05; (2) ** indicates level of significance at *p*<0.01; (3) the significance codes apply to comparisons to the control treatment only.

According to the KOG and KEGG pathway analyses on all differentially expressed genes, 10 genes with significantly differential expression (*p*<0.05; |log_2_(fold-change)| ≥10) were screened out: *ubr5*, *arhgef7a*, *mcamb*, *slc12a8*, *atp6v0ca*, *esyt3*, *mark2a*, *appb*, *macf1* and *ralgapa1*. They were associated with immune and nervous systems, and Table 1 lists their information in detail. We further explored the differences of zebrafish gene transcripts in different DKAs-exposure treatments, and list the significantly differential transcripts in Table 1 and S3 Table. These transcripts resulted from alternative splicing, some of them not giving rise to a protein. The screened 10 genes from the control and treatment groups were validated by qRT-PCR to confirm the consistency between transcriptome and transcription levels, using the signal of *β-actin* as a stable reference gene. Results showed that 7 genes showed to be consistent between qRT-PCR and RNA-seq (Fig 4). The gene expression of *arhgef7a*, *mcamb*, *mark2a*, *appb*, *macf1* and *ralgapa1* was up-regulated in the 6.25 mg/L treatment group, while down-regulated in 12.5 mg/L treatment group when compared to the control group. Down-regulation of *ubr5* and *esyt3*, *and* up-regulation of *slc12a8* and *atp6v0ca* were observed in both 6.25 and 12.5 mg/L treatment groups.

**Table 1:** Information on 10 screened differentially expressed genes.

Gene	Description	KEGG	Locus	Transcript ID	log_2_(fold-change)		*p*-value
					6.25/Con	12.5/Con	
arhgef7a	Rho guanine nucleotide exchange factor (GEF) 7a	Regulation of actin cytoskeleton	chr9:22569229–22600300	ENSDART00000141408-C	-16.60		0.04
						-16.60	0.01
esyt3	extended synaptotagmin-like protein 3	NA	chr9:24817196–24840377	ENSDART00000027443-C	11.70		0.01
				ENSDART00000129737-NC		-16.60	0.05
slc12a8	solute carrier family 12 (potassium/chloride transporters), member 8	NA	chr9:39697284–39734546	ENSDART00000110651-C	17.00		0.05
				ENSDART00000135774-C		17.30	0.03
appb	amyloid beta (A4) precursor protein b	NA	chr9:35997607–36041142	ENSDART00000077908-C	18.80		0.01
						20.90	<0.01
				ENSDART00000122679-C	20.60		0.02
				ENSDART00000077901-C		-13.40	<0.01
mark2a	MAP/microtubule affinity-regulating kinase 2a	NA	chr21:26598188–26668126	ENSDART00000143239-C	19.50		0.01
				ENSDART00000040754-C		19.00	0.02
mcamb	melanoma cell adhesion molecule b	NA	chr15:22625409–22696300	ENSDART00000020425-C	-16.60		0.04
				ENSDART00000115362-C		-16.60	0.04
macf1	microtubule-actin crosslinking factor 1	NA	chr19:36588200–36899421	ENSDART00000146394-C	18.00		0.01
				ENSDART00000054274-C		-9.95	0.02
ralgapa1	Ral GTPase activating protein, alpha subunit 1 (catalytic)	NA	chr17:12630934–12787578	ENSDART00000154984-C	-16.60		0.05
						-16.60	0.05
atp6v0ca	ATPase, H+ transporting, lysosomal, V0 subunit c, a	Oxidative phosphorylation; Lysosome; Phagosome	chr3:15664660–15682998	ENSDART00000023859-C	-16.60		0.02
				ENSDART00000123621-C		13.70	<0.01
ubr5	ubiquitin protein ligase E3 component n-recognin 5	Ubiquitin mediated proteolysis	chr16:56147790–56252683	ENSDART00000134444-C	16.30		<0.01
				ENSDART00000128437-C	-16.60		0.05
						-16.60	0.03

Note: (1) In the fifth column of Table 1, “C” following each transcript indicates that this transcript can be translated into protein; (2) “NC” following each transcript indicates that this transcript can not be translated into protein.

### Lysozyme activity and AKP, C3 and IgM concentration assays

Biomarkers analysis and histopathological observation were used to detect physiological effects of DKAs exposure. Biomarkers indicate biochemical indicators that can label the changes in structures or functions of system, organ, tissue, cell or subcells. They are signal indicators showing abnormal effects of environmental pollutants at the different biological (molecular, cellular and individual) levels before being severely damaged, and can provide early warning of serious toxic damage. The level of antibody in serum can reflect the body’s immune capacity, and thus it is necessary to analyze the antibody’s content in serum for sake of assessing the level of fish immunity. The complement system is an important part of the immune system, which protects the body from invasion of various microorganisms by mediating cell lysis, inflammatory reaction, clearance of immune complexes, immune regulation as well as removal of differentiated cells. In complement proteins, Complement 3 (C3) is the core of complement system, which is the highest in blood. AKP activity is not only a comprehensive reflection of cellular and humoral immunity, but also an indicator for measuring immune function and body state, which shows the body’s defense against the infection of exogenous microorganisms. Lysozyme is an important part of the non-specific immune system in fish, which can be used as an important indicator reflecting the function of immune system. AKP, IgM, lysozyme and C3 are good criteria for measuring immune functions. Changes in concentrations of C3 and immunoglobulin IgM in zebrafish play an important role in antigen-antibody reaction. The concentration of C3 was significantly (*p* ≤0.01) decreased in both treatments compared to control; however, a concentration-gradient effect was not observed (i.e., no significant difference between 12.5 and 25 mg/L treatments). IgM concentration (0.045 g/L) did not change between the 12.5 mg/L treatment and control. However, at the 25 mg/L DKAs concentration, zebrafish produced a higher concentration of IgM (0.068 g/L). In contrast, when DKAs concentrations reached 50 mg/L, IgM concentration was significantly reduced (0.001 g/L).

**Fig 5:**
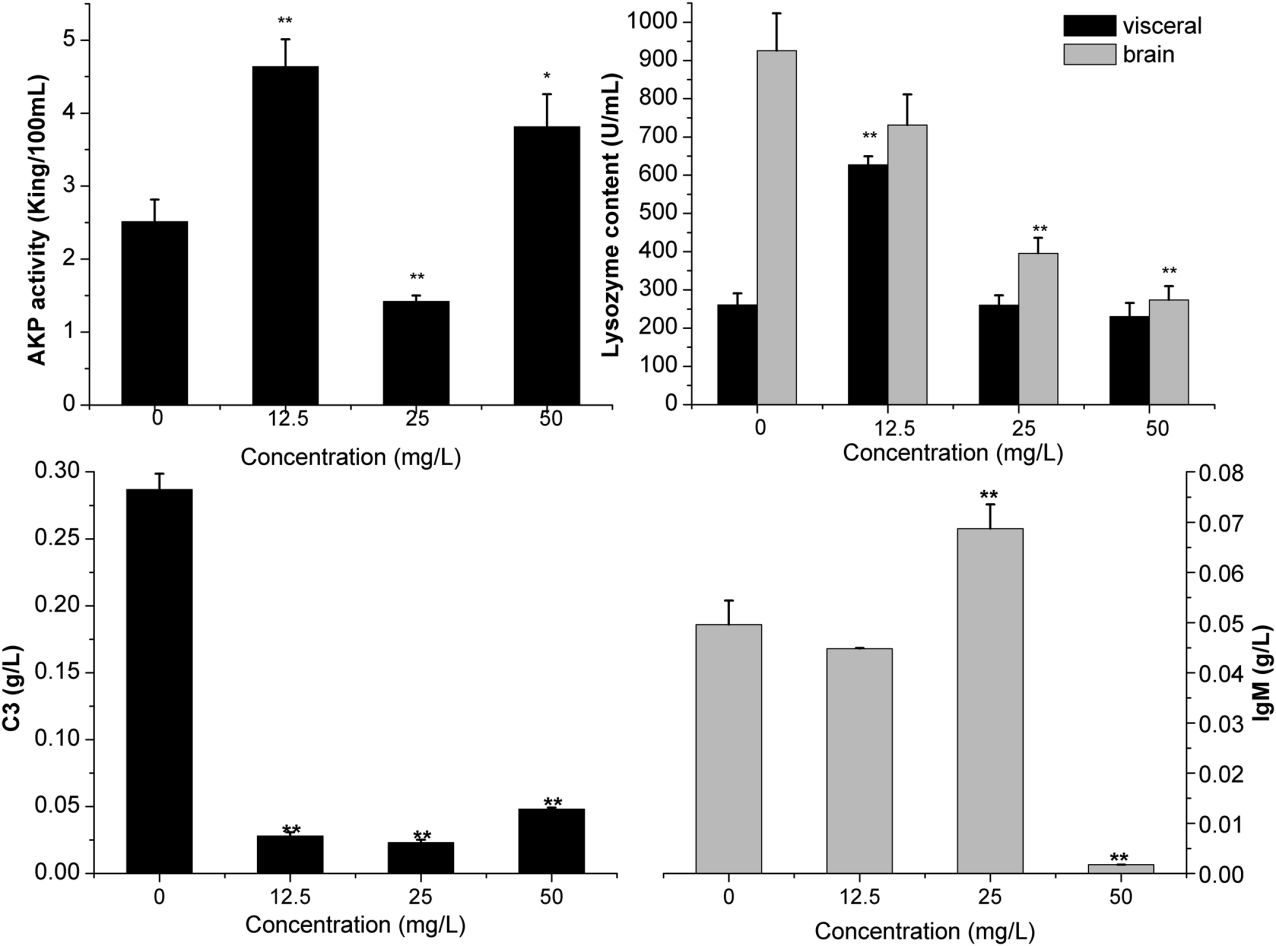
Effects of DKAs concentrations on AKP activity, and contents of lysozyme, C3 and IgM. Note: (1) * indicated level of significance at *p*<0.05; (2) ** indicated level of significance at *p*<0.01; (3) the significance codes apply to comparisons to the control treatment only.

Additionally, we measured the concentration of lysozyme in viscera and brain, and AKP activity in blood. Lysozyme concentration in controls differed significantly between brain and viscera, and their responses to DKAs-exposure treatments were also different. For the 12.5 mg/L treatment, lysozyme concentration in the brain was slightly decreased, but it was increased significantly in the viscera. For the 25 and 50 mg/L treatments, lysozyme concentrations in the viscera had no changes, however, those in the brain were significantly decreased, especially for the 50 mg/L treatment. The AKP activity was significantly increased in the 12.5 and 50 mg/L treatments, but inhibited in the 25 mg/L treatment. Namely, AKP activity changed irregularly with increasing DKAs concentrations and warrant further investigation to explain this response (Fig 5).

### Histopathological observation

**Fig 6:**
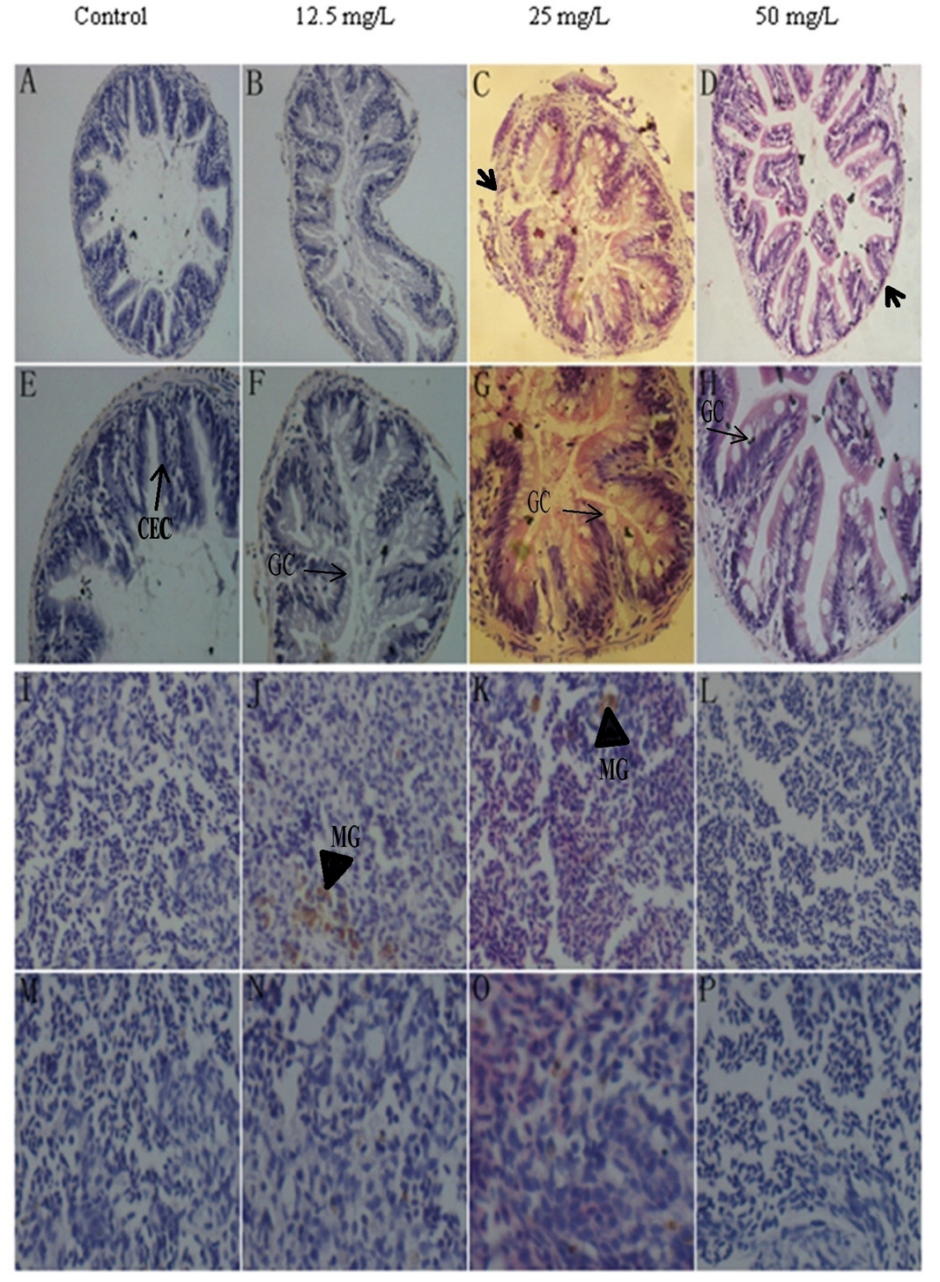
HE dyeing of intestine and spleen. Note: A-D, Intestinal 200×; E-H, Intestinal 400×; I-L, Spleen 200×; M-P, Spleen 400×. CEC: columnar epithelial cells; GC: goblet cells; MG: metachromatic granules.

Under 200× magnification, the small intestine structure in the control group was complete and clear, and its peripheral muscle layer was relatively intact. In comparison, rupture and dissolution phenomena were observed in DKAs treatments. This was especially the case for the 50 mg/L treatment where the intestinal wall was thin and the muscular layer almost invisible (Fig 6A–6D). At 400× magnification, the number of goblet cells increased sharply in intestinal villi of DKAs-exposure groups, the columnar epithelial cells of intestinal villi became swelled, and the nucleus became slender (Fig 6E–6H). Spleen cells in the control group examined under 200× magnification were uniformly distributed and its tissue structure was normal. However, spleen cells were unevenly distributed, tissue structure was seriously damaged in 25 and 50 mg/L treatments, and numerous brown metachromatic granules occurred in 12.5 and 25 mg/L treatments (Fig 6I–6L). At 400× magnification, many cells became vague and indistinct, and the nucleus became larger in the 12.5 and 25 mg/L treatments compared to control. This phenomenon may be explained by the occurrence of DKAs-induced inflammation. As for the 50 mg/L treatment, the number of spleen cells was distinctly scarce (Fig 6M–6P).

### TEM ultramicrotomy for intestine, spleen and gills

**Fig 7:**
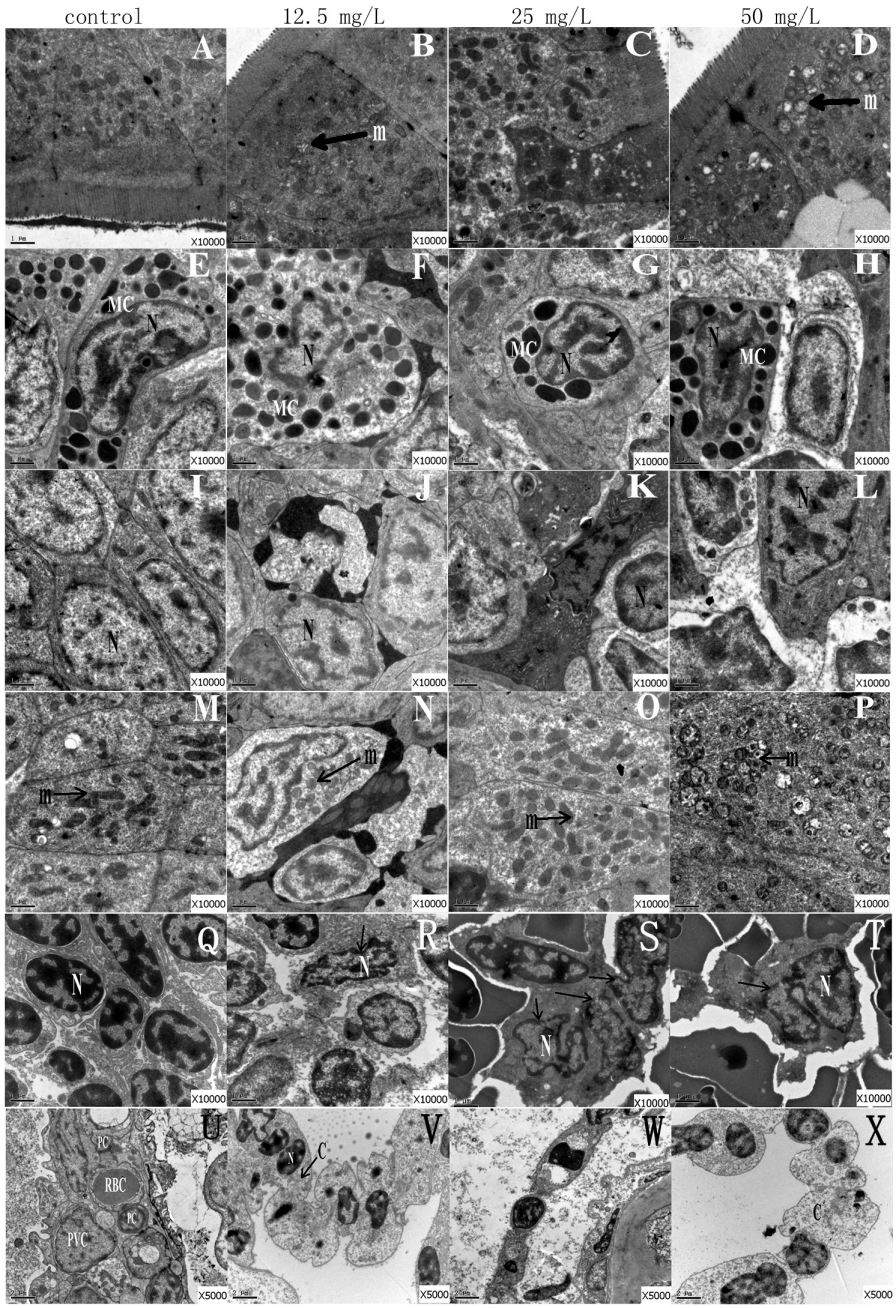
TEM images of zebrafish intestine, spleen and gill tissues. Note: A-D, Columnar epithelial cells; E-H, Mast cell; I-L, Intestinal epithelial cell; M-P, Mitochondrial; Q-T, the dendritic cells in spleen; U-X, cells in gill. N: nuclear; m: mitochondria; MC: mast cells; RBC: red blood cell; PC: pillar cell; PVC: flat epithelial cell; C: cytoplasm.

Zebrafish at 90 dpf were selected to investigate their histopathological changes after exposure to a series of DKAs concentrations (0, 12.5, 25 and 50 mg/L). As shown in Fig 7, cytoplasm and mitochondria in columnar epithelial cells were distributed uniformly in the control group. For the 12.5 mg/L treatment, no obvious changes in cytoplasm were observed, but swelling and vacuolization of mitochondria occurred. A close connection between cells, dense and clear mitochondria, and slight dissolution of cytoplasm was observed in the 25 mg/L treatment. Additionally, some cells became darker and condensed. For the 50 mg/L treatment, although cytoplasm did not obviously change, vacuolization and edema appeared in mitochondria compared to the control group (Fig 7A–7D).

Cytoplasm, nucleus chromatin and particles in mast cells were dense and uniformly distributed in the control group. The cytoplasm and nucleus in mast cells displayed obvious edema in the 12.5 mg/L treatment, while the cytoplasm showed slight edema with no prominent changes to the nucleus of mast cells in the 25 mg/L treatment. Exposure to 50 mg/L DKAs induced no obvious morphological changes of mast cells, but their surrounding cells demonstrated serious edema and cytoplasm dissolution (Fig 7E–7H).

Intestinal epithelial cells in the control group had regular shape, cell membranes were clearly visible, and cytoplasm and nuclear chromatin were uniformly distributed. In contrast, the shapes of epithelial cells in the three DKAs-exposure groups were irregular. The 25 and 50 mg/L treatments displayed severe deformation of epithelial cells, swelling of cytoplasm and nuclear chromatin. In comparison, while apoptosis and swelling of cells did not occurred, pyknotic nuclei did appear in the 12.5 mg/L treatment. Cell karyopyknosis, swelling and dissolution of cytoplasm occurred in the 50 mg/L treatment (Fig 7I–7L). Mitochondrial cristae was clear in the control group, while the lighter mitochondrial color, slight edema and blurred cristae appeared in the 12.5 and 25 mg/L treatments. Further, mitochondrial edema and even vacuolization occurred in the 50 mg/L treatment (Fig 7M–7P).

In control group, the dendritic cells in spleen have clearly round or oval nuclei, clear cellular membrane and uniformly distributing cytoplasm, and unambiguous boundary among cells. In contrast, unclear nucleoli, irregular nucleus shape, slight dissolution of chromatin and swelling of cytoplasm occurred in 12.5 mg/L DKAs treatment. Some dentritic cell nuclei were unclear, the shape of nuclei was irregular, the chromatin was obviously dissolved, the cell membrane was not clearly visible and no distinct boundary existed among cells in 25 mg/L DKAs treatment. Especially in 50 mg/L DKAs exposure, dentritic cell nucleoli almost disappeared, the nucleus shape was irregular, the cell membrane was not clear and the cell pseudopodium was fractured (Fig 7Q–7T).

Blood channels, flattened epithelial cells and supporting cells were clearly visible in zebrafish gills of the control group. Nuclei in supporting cells were obvious, which filled the entire cell, and also its chromatin was uniformly distributed. For flatten epithelial cells, cytoplasm and nucleus chromatin was evenly and sinusoidal distributed between flatten epithelial cells, and supporting cells were clearly visible. For the three DKAs-exposure treatments, boundaries among cells were not clear and there was the occurrence of exfoliated epithelial cells, dissolution of red blood cells and blood vessels, and karyopyknosis of supporting cell nuclei. Especially, severe swelling and dissolution of cytoplasm in supporting cells occurred in the 12.5 and 50 mg/L treatment groups (Fig 7U–7X).

## Discussion

Prior to extraction of total RNA, we performed simple anatomy of the whole fish, i.e., removing gill, fin and tail tissue, while retaining visceral mass, head, spine and other nerve systems. The main reason for this simple anatomy was that under the circumstances of unknown DKAs target, we tried to avoid missing the target genes with specific expression in some organs. Except for specific target organs, the previous literatures reported the application of whole fish for RNA-seq [17–19].

Protein classification by KOG revealed that a significant function for proteins regulated by the differentially expressed genes was signal transduction. Yin et al. documented a global gene profile for mouse liver following acute tetracycline treatment, as determined on oligonucleotide microarrays, and they also found that the largest groups of gene products affected by tetracycline exposure were those involved in signal transduction, nucleic acid metabolism, developmental, and protein metabolism [5]. In studying toxic chemical-altered lipid metabolism in mouse liver slices by transcriptome analysis, Szalowska et al. found that tetracycline obviously down-regulated functional clusters related to inflammation and apoptosis [20]. In this investigation, KEGG pathway enrichment analyses showed that the differentially expressed genes were involved in MAPK, neuroactive ligand-receptor, focal adhesion, endocytosis, and regulation of actin cytoskeleton pathways. Most of the pathways with high enrichment were found to be involved in nervous and immune systems, and we decided to focus our subsequent analysis on immune functions.

In the 10 significantly differential genes, 7 genes showed the consistency between qRT-PCR and RNA-seq, among which 5 genes were immune-related, and the differential expression by these genes may result in immune dysfunction. *Mcamb* (also known as CD146) is highly expressed by vascular endothelial cells, smooth muscle cells and pericytes that are elements of the blood vessel wall. CD146 is a biomarker for endothelial cell function [21] and plays an important role in mediating intercellular adhesion, regulating the cell permeability, promoting endothelial cell proliferation, and participating in cell transmembrane signal transduction [22–24]. CD146 T cells have been shown to produce IL-17 [25], which can induce cell inflammatory cytokines and chemokines. *Mcamb* can also promote transfer of pigments [26–27] and prostate cancer cells [28]. Shih and coworkers [29] proposed that the expression of *mcamb* inhibited generation of breast cancer, and that its over-expression in MCF-7 suppressed tumor generation in SCID mice.

A progestin-induced protein belonging to the HECT family was encoded by u*br5*. The HECT family proteins play a part as E3 ubiquitin-protein ligases for ubiquitin-mediated proteolysis. The *ubr5* gene potentially participates in the regulation of cell proliferation or differentiation, while its expression is easily interrupted by a variety of cancers. Ubiquitin ligase E3 through regulating the ubiquitin protein is involved in a variety of physiological processes in the cell. Interruption of the ubiquitin pathway may result in difficulty in regulating the cell cycle, which may causes cancer in certain cells, tissues and organs [30].

*MARKs* can regulate microtubule-binding proteins, MAPtau, in the nervous system, which maintain cell microtubule network stability through disassociation of tau from microtubules. As a member of the AMPK-associated kinase family, *MARKs* regulate the stability of microtubule and cellular polarity, while also functioning in reproduction and balancing of the immune system and body’s energy system. For example, *MARK2* in fatty liver plays an important role in regulating the balance of glucose, which in turn regulates reproduction and immune system balance, learning/memory ability, and metabolism of glucose and energy. As a result, *MARK2*-knockout mice had reproductive defects, imbalance in the immune system, weak learning/memory ability, growth retardation, metabolic disorders, and a variety of other symptoms [31–34]. IGF-1 levels in serum of *MARK2*-knockout mice decreased, which may explain the stunting phenomenon in these mice. *MARK2*-knockout mice displayed a smaller size and their adipose tissue was distributed asymmetrically [34]. Driessen et al. studied the applicability of whole zebrafish embryo model (ZFE) for hepatotoxicity testing by combining next generation sequencing-based gene expression profiling, and demonstrated that the high concordance in hepatotoxicity pathways between the zebrafish embryos and mouse liver implied similar regulation at the level of transcription factors [35]. The transcriptional changes on single gene were also reported by Driessen et al., who proved that ZFE model allowed for identification of hetatotoxicants without discrimination into specific phenotypes [36].

In summary, DKAs-exposure treatments affected some immune-related gene expression at the transcriptional level, and most of the immune-related genes affected by DKAs participated in conventional signaling pathways in cells, such as MAPK, insulin, mTOR and PI3K-Akt signaling pathways. As a result, DKAs exposure to zebrafish could lead to abnormal cell signaling pathways, changes of cell morphology, and deterioration of cell viability. The above-mentioned adverse effects could cause serious disorders in immune system function, occurrence of inflammation, allergic reaction, and even tumor formation.

The enhanced IgM levels may lead to an increase of antigen-antibody complexes, which is an activator of the complement classical pathway. As a result, excessive activation of enzyme-catalyzed reactions may occur in the complement classical pathway (C1-C9), which causes excessive consumption of complement C3 and decreasing C3 content in blood. For the 50 mg/L DKAs treatment, both the concentrations of IgM and C3 decreased in blood, which may be related to disorders in primary synthetic organs, such as spleen and liver damage. These phenomena were confirmed by histopathological and TEM observations, which were also in agreement with Wang and coworkers [37] findings that DKAs exposure resulted in serious liver damage to zebrafish. The previous studies also demonstrated that tetracycline could induce zebrafish liver lipid accumulation as observed with oil-red-O staining [35–36].

An increase of AKP activity in the 12.5 and 50 mg/L DKAs treatments may result from AKP entering the blood from the lymphatic channel and hepatic sinus due to liver damage/dysfunction, or from skeletal disorders [17]. On the contrary, a decrease of AKP activity in the 25 mg/L treatment may result from DKAs-inhibited activities of neutrophils and phagocytes, which reduces AKP secretion capacity, or alternatively from DKAs-induced chronic nephritis, anemia or thyroid function. Whether these phenomena resulted from direct action of DKAs or from indirect impact on other immune factors requires further investigation.

Lysozyme is one of the important nonspecific immune factors in leucocytes, whose immune function is exerted by destroying the cell walls of gram positive bacteria. When organisms meet bacterial infectious diseases or other stimulus factors, changes in lysozyme activity may enhance immune defenses. The content of lysozyme was higher in the brain than in the visceral mass, but it remained unchanged in the brain for the 12.5 mg/L DKAs treatment. However, the 25 and 50 mg/L DKAs treatments led to a significant decrease in lysozyme content. This phenomenon may be associated with the blood-brain barrier, which indicates the structure of certain substances into cerebral circulation blood in cerebral capillary tissues. This kind of structure can make brain tissue less affected by the harmful substances in blood circulation so as to maintain the basic stability of brain tissue environment. The molecules of fluoroquinolones, due to fat solubility from fluorine atoms bearing in their molecular structures, easily break through blood-brain barrier and enter into brain tissues, which lead to increase in excitability of central nervous system and extensive necrosis of brain tissues [38].

Fish gill is the organ for gas exchange with outside environment, its filamentous surface is full of tiny blood vessels, and the water-dissolved oxygen can enter into the blood by blood vessels for respiration. By TEM observations, we found that DKAs exposure led to shedding of gill filamentous epithelial cells, and deformation or disappearance of red blood cells. These cellular structural abnormalities possibly caused that the oxygen molecules could not be fully combined with hemoglobin in red blood cells. Lacking of oxygen may cause damage of zebrafish organs, deactivitation of enzymes and metabolic disorders. Additionally, many metachromatic particles were found in zebrafish spleen after DKAs exposure. Thus, the immune functional disorders could result from damage of zebrafish immune organs such as small intestine and spleen due to DKAs exposure [39].

## Conclusion

Based on transcriptome data, we found that there were 106 co-differentially expressed genes in three mutual comparison groups (control, 6.25 and 12.5 mg/L treatment). After functional annotation and metabolic pathway analysis, 10 genes were identified among which 9 of them showed consistency between RNA-seq and qRT-PCR. Detection of immune-related biomarkers (C3, lysozyme, IgM and AKP) and histopathological observation further corroborated that long-term (90 days) chronic DKAs exposure resulted in abnormal expression of immune genes and enzymes, and variable levels of damage to immune-related organs. These complex effects may lead to zebrafish immune system dysfunction and occurrence of diseases concerned with the immune system.

## Statements

The primary data on sequencing have been uploaded at http://www.ncbi.nlm.nih.gov/sra/, and the accession number is SRP070923.

## Supporting Information

S1 Fig Chemical structures for each DKA compound. (DOC)

S2 Fig HPLC profiles of six DKAs. (DOC)

S3 Fig Cluster analysis of 106 co-differentially expressed genes. (DOC)

S1 Table Primers used for qRT-PCR expression analysis. (DOC)

S2 Table Sequencing result quality, statistics of reference genome in comparison with reads, and region and interval distribution of differential gene expression value in each sample. (DOC)

S3 Table Information on 106 co-differentially expressed genes. (DOC)

S4 Table The mostly enriched KEGG pathway of the differentially expressed genes. (DOC)
