# Supplementary figures and images for: Immunotoxicity of β-Diketone Antibiotic Mixtures to Zebrafish (Danio rerio) by Transcriptome Analysis
Source: PLoS One. 2016 Apr 5;11(4):e0152530. doi: 10.1371/journal.pone.0152530 (PMC4821563; doi:10.1371/journal.pone.0152530)

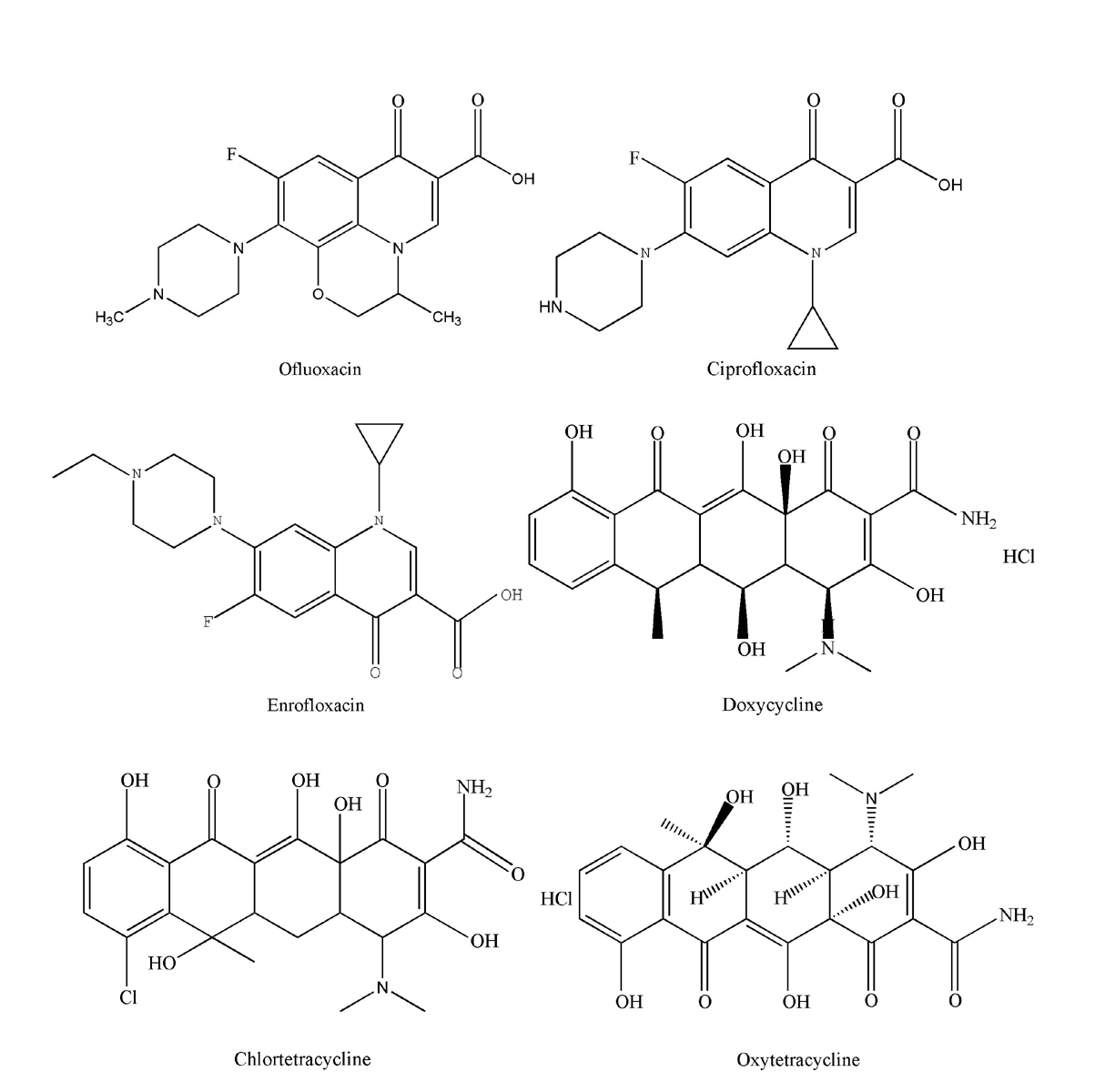


**S1 Fig .** Chemical structures for each DKA compound

Supplement: S1 Fig — (DOC) [file pone.0152530.s001.doc]
