# Supplementary material for: Immunotoxicity of β-Diketone Antibiotic Mixtures to Zebrafish (Danio rerio) by Transcriptome Analysis
Source: PLoS One. 2016 Apr 5;11(4):e0152530. doi: 10.1371/journal.pone.0152530 (PMC4821563; doi:10.1371/journal.pone.0152530)
Supplement: S2 Fig — (DOC) [file pone.0152530.s002.doc]

**S2 Fig.** HPLC profiles of six DKAs

Note: (1) “a”, chromatograms for six DKAs after 24-h DKAs exposure in 6.25 mg/L treatment group; (2) “b”, chromatograms for six DKAs at an initial fortification level of 6.25 mg/L; and (3) peaks 1-6 indicate doxycycline, chlortetracycline, ofloxacin, oxytetracycline, ciprofloxacin and enrofloxacin, respectively.
