# Supplementary material for: Immunotoxicity of β-Diketone Antibiotic Mixtures to Zebrafish (Danio rerio) by Transcriptome Analysis
Source: PLoS One. 2016 Apr 5;11(4):e0152530. doi: 10.1371/journal.pone.0152530 (PMC4821563; doi:10.1371/journal.pone.0152530)
Supplement: S3 Fig — (DOC) [file pone.0152530.s003.doc]

**
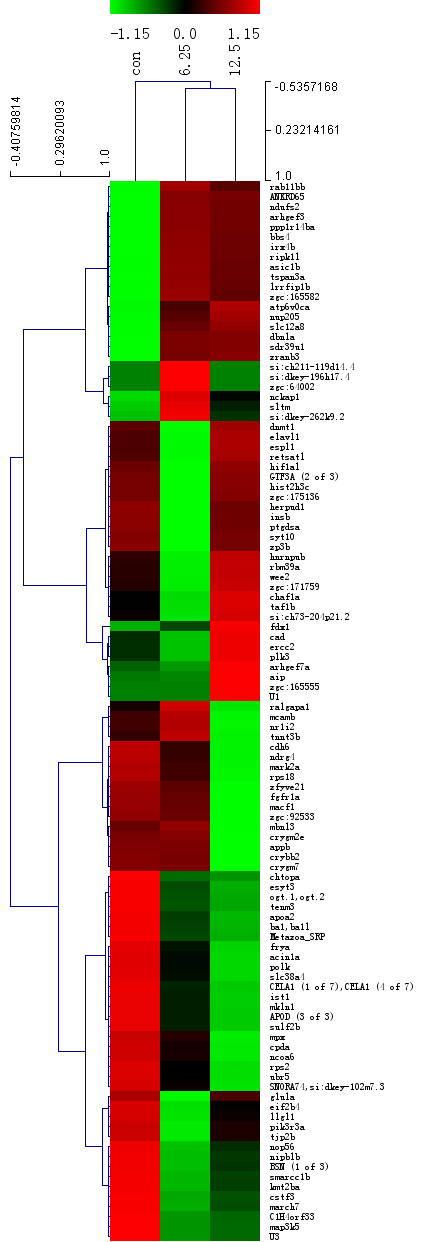
**

**S3 Fig.**Cluster analyses of 106 common differential expression genes

Note: Green represents low expression and red represents high expression.
