# Supplementary material for: Immunotoxicity of β-Diketone Antibiotic Mixtures to Zebrafish (Danio rerio) by Transcriptome Analysis
Source: PLoS One. 2016 Apr 5;11(4):e0152530. doi: 10.1371/journal.pone.0152530 (PMC4821563; doi:10.1371/journal.pone.0152530)
Supplement: S1 Table — (DOC) [file pone.0152530.s004.doc]

**S1 Table.** Primers used for qRT-PCR expression analysis

| Gene | Primer | Sequence (5’-3’) | Product size |
| --- | --- | --- | --- |
| β-actin | β-actin-F | GCGGAAACTGGCAAAGGG | 109 |
|  | β-actin-R | AGGGCAAAGTGGTAAACG |  |
| ESYT3 | ESYT3-F | TGAGTAACGGGGTCAACGA | 134 |
|  | ESYT3-R | ATACACGGGGTAGCAGATGG |  |
| LRRFIP1b | LRRFIP1b-F | CTTTATTCCTCCGGTTTGACG | 119 |
|  | LRRFIP1b-R | TGACAGTTTGCTGATGCGAC |  |
| MAP3K5 | MAP3K5-F | GGCACCTTTGGAGTTGTCTA | 148 |
|  | MAP3K5-R | CGATGTTCTTGTGCTTGAGG |  |
| MARK2a | MARK2a-F | GGGCTCTTTACAACTTGGACT | 160 |
|  | MARK2a-R | TGGATGGCTAAAAGGGGTAT |  |
| MCAMb | MCAMb-F | CTGCTATGCACAAGGCTACC | 159 |
|  | MCAMb-R | TTGGCAATGAGATCAGAGGTG |  |
| ARHGEF7a | ARHGEF7a-F | AGCAACATCACGGAGTTTCTG | 148 |
|  | ARHGEF7a-R | CGCTGCCAATGCCAATA |  |
| UBR5 | UBR5-F | CACAACAGGAGAACCGAAAG | 108 |
|  | UBR5-R | CAGGCTGGTAAAACAGAGGA |  |
| TSPAN3a | TSPAN3a-F | GCGAAGGTAGAAGATGAGGTC | 142 |
|  | TSPAN3a-R | AGTCTGCGAAGTTGGTGATG |  |
| SYT10 | SYT10-F | TCCCTTTGCTATCTTCCCACG | 139 |
|  | SYT10-R | TCTTCAGCCTCCGACCATCA |  |
| PLK3 | PLK3-F | GATGTTTGGTCGCTGGGTT | 130 |
|  | PLK3-R | GAGTGAGGGATGAAGGAAGG |  |
| PIK3R3a | PIK3R3a-F | AGGAACAACGGCACGAACA | 179 |
|  | PIK3R3a-R | TTACAGTGGGTCACGGGATA |  |
| NDRG4 | NDRG4-F | CTCGGAATGTAGGGCAAAT | 88 |
|  | NDRG4-R | GGGCGACTGGAAGTATCTGT |  |
| MPX | MPX-F | GCCTTCACATCCCACATAGC | 199 |
|  | MPX-R | TGTTCTCAGGGGTGCCATA |  |
