# Supplementary material for: Immunotoxicity of β-Diketone Antibiotic Mixtures to Zebrafish (Danio rerio) by Transcriptome Analysis
Source: PLoS One. 2016 Apr 5;11(4):e0152530. doi: 10.1371/journal.pone.0152530 (PMC4821563; doi:10.1371/journal.pone.0152530)
Supplement: S2 Table — (DOC) [file pone.0152530.s005.doc]

**S2 Table.** Sequencing result quality, statistics of reference genome in comparison with reads, and region and interval distribution of differential gene expression value in each sample

| Sample  (mg/L) | **Sequencing result quality** | | | | | | | **Reference genome in comparison with reads** | | | **Reference genome in comparison with region** | | | **Interval distribution of different gene expression value** | | | | | |
| --- | --- | --- | --- | --- | --- | --- | --- | --- | --- | --- | --- | --- | --- | --- | --- | --- | --- | --- | --- |
|  | Raw data  read | Base | Valid data  read | Base | Valid %  read | Q30% | GC % | Mapped reads | Unique Mapped reads | Multi Mapped reads | Exon | Intron | Intergenic | 0-0.1 FI | 0.1-0.3 FI | 0.3-3.57 FI | 3.57-15 FI | 15-60 FI | >60 FI |
| Control | 72349790 | 9.04 G | 71532662 | 8.94 G | 98.87 | 96.24 | 48 | 27752966(38.8%) | 25742103(36.0%) | 2010863  (2.8%) | 89.2% | 7.7% | 3.1% | 63512  (72.0%) | 1912  (2.2%) | 6629  (7.5%) | 6598  (7.5%) | 4880  (5.5%) | 4686  (5.3%) |
| 6.25 | 66250226 | 8.28 G | 65426852 | 8.18 G | 98.76 | 95.61 | 46.50 | 36032694(55.1%) | 28195816(43.1%) | 7836878  (12.0%) | 87.1% | 7.9% | 3.8% | 48624  (55.1%) | 1759  (2.0%) | 9675  (11.0%) | 12013  (13.6%) | 5530  (6.3%) | 10616  (12.0%) |
| 12.5 | 85071104 | 10.63 G | 84020924 | 10.50 G | 98.77 | 87.29 | 44.50 | 45610683(54.3%) | 41209033(49.1%) | 4401650  (5.2%) | 88.9% | 7.9% | 3.3% | 46509  (52.7%) | 1606  (1.8%) | 10796  (12.2%) | 12399  (14.1%) | 5978  (6.8%) | 10929  (12.4%) |

Note: Raw data read: initial sequence reads quantity; Valid data read: reads quantity after quality check and trimming; Q30: percent of reads with accuracy rate over 99.9%; FI=FPKM interval.
