# Supplementary material for: Immunotoxicity of β-Diketone Antibiotic Mixtures to Zebrafish (Danio rerio) by Transcriptome Analysis
Source: PLoS One. 2016 Apr 5;11(4):e0152530. doi: 10.1371/journal.pone.0152530 (PMC4821563; doi:10.1371/journal.pone.0152530)
Supplement: S3 Table — (DOC) [file pone.0152530.s006.doc]

**S3 Table. Information on 106 differentially expressed** genes

| Gene | Description | KEGG | Locus | Transcript ID | log_2_(fold_change) | | p-value |
| --- | --- | --- | --- | --- | --- | --- | --- |
|  |  |  |  |  | 6.25/Con | 12.5/Con |  |
| acin1a | apoptotic chromatin condensation inducer 1a | Spliceosome | chr2:37900497-37932766 | ENSDART00000146485-C | 18.20 |  | 0.04 |
|  |  |  |  | ENSDART00000136082-C |  | -15.70 | <0.01 |
| aip | aryl hydrocarbon receptor interacting protein | NA | chr1:40343978-40401983 | ENSDART00000143786-NC | -18.30 |  | <0.01 |
|  |  |  |  |  |  | -16.60 | <0.01 |
| ANKRD65 | ankyrin repeat domain 65 | NA | chr18:39836151-39850284 | ENSDART00000128495-C | 17.90 |  | 0.02 |
|  |  |  |  | ENSDART00000098671-C |  | 17.40 | 0.02 |
| apoa2 | apolipoprotein A-II | NA | chr16:26218270-26219647 | ENSDART00000132689-NC | 1.31 |  | 0.04 |
|  |  |  |  |  |  | -16.60 | <0.01 |
| APOD (3 of 3) | apolipoprotein D | NA | chr2:36928293-36936822 | ENSDART00000098463-C | 13.40 |  | <0.01 |
|  |  |  |  |  |  | 13.60 | <0.01 |
|  |  |  |  | ENSDART00000138952-C |  | -16.60 | 0.01 |
|  |  |  |  | ENSDART00000143468-C |  | -16.60 | 0.02 |
| appb | amyloid beta (A4) precursor protein b | NA | chr9:35997607-36041142 | ENSDART00000077908-C | 18.80 |  | 0.01 |
|  |  |  |  |  |  | 20.90 | <0.01 |
|  |  |  |  | ENSDART00000122679-C | 20.60 |  | 0.02 |
|  |  |  |  | ENSDART00000077901-C |  | -13.40 | <0.01 |
| arhgef3 | Rho guanine nucleotide exchange factor (GEF) 3 | NA | chr22:41997099-42038629 | ENSDART00000025482-C | 17.40 |  | 0.04 |
|  |  |  |  | ENSDART00000130305-C |  | 16.70 | 0.04 |
| arhgef7a | Rho guanine nucleotide exchange factor (GEF) 7a | Regulation of actin cytoskeleton | chr9:22569229-22600300 | ENSDART00000141408-C | -16.60 |  | 0.04 |
|  |  |  |  |  |  | -16.60 | 0.01 |
| asic1b | acid-sensing (proton-gated) ion channel 1b | NA | chr22:6991901-7206812 | ENSDART00000028603-C | 18.30 |  | <0.01 |
|  |  |  |  | ENSDART00000140236-C |  | 16.70 | 0.03 |
| atp6v0ca | ATPase, H+ transporting, lysosomal, V0 subunit c, a | Oxidative phosphorylation;  Lysosome;  Phagosome | chr3:15664660-15682998 | ENSDART00000023859-C | -16.60 |  | 0.02 |
|  |  |  |  | ENSDART00000123621-C |  | 13.70 | <0.01 |
| ba1 | ba1 globin | NA | chr3:55950128-55957638 | ENSDART00000142014-NC | -12.40 |  | 0.01 |
|  |  |  |  |  |  | -16.60 | <0.01 |
|  |  |  |  | ENSDART00000145522-NC |  | 17.20 | 0.02 |
| bbs4 | Bardet-Biedl syndrome 4 | NA | chr25:2608099-2642154 | ENSDART00000139517-NC | 20.30 |  | <0.01 |
|  |  |  |  | ENSDART00000092919-C |  | 17.70 | 0.02 |
| BSN (1 of 3) | bassoon presynaptic cytomatrix protein | NA | chr11:38354558-38372994 | ENSDART00000127184-C | 16.90 |  | 0.03 |
|  |  |  |  | ENSDART00000109753-C |  | 17.30 | 0.02 |
| C1H4orf33 | chromosome 4 open reading frame 33 | NA | chr1:11660586-11694017 | ENSDART00000134575N-C | -16.60 |  | 0.04 |
|  |  |  |  | ENSDART00000138622-C |  | 19.90 | 0.02 |
| cad | carbamoyl-phosphate synthetase 2, aspartate transcarbamylase, and dihydroorotase | Pyrimidine metabolism;  Alanine, aspartate and glutamate metabolism | chr20:38813027-38852397 | ENSDART00000061365-C | -16.60 |  | 0.05 |
|  |  |  |  |  |  | -16.60 | 0.02 |
|  |  |  |  | ENSDART00000075262-C |  | -16.60 | 0.04 |
|  |  |  |  | ENSDART00000153310-C |  | 13.00 | 0.05 |
| cdh6 | cadherin 6 | NA | chr2:28216906-28270171 | ENSDART00000131506-C | -16.40 |  | <0.01 |
|  |  |  |  | ENSDART00000013638-C | 12.90 |  | <0.01 |
|  |  |  |  |  |  | 12.10 | 0.02 |
| CELA1 (1 of 7) | chymotrypsin-like elastase family, member 1 | NA | chr22:7477352-7521632 | ENSDART00000143159-NC | -4.74 |  | 0.03 |
|  |  |  |  |  |  | -16.60 | 0.02 |
| chaf1a | chromatin assembly factor 1, subunit A (p150) | NA | chr22:22639306-22665315 | ENSDART00000142027-C | -11.10 |  | 0.01 |
|  |  |  |  |  |  | -11.60 | <0.01 |
|  |  |  |  | ENSDART00000089534-C |  | 14.90 | 0.02 |
|  |  |  |  | ENSDART00000111711-C |  | -16.60 | 0.04 |
| chtopa | chromatin target of PRMT1a | NA | chr19:24383330-24403801 | ENSDART00000079842-C | 20.70 |  | <0.01 |
|  |  |  |  |  |  | 20.60 | <0.01 |
| cpda | carboxypeptidase D, a | NA | chr15:24110407-24148158 | ENSDART00000156424-C | 19.10 |  | 0.03 |
|  |  |  |  |  |  | 17.70 | 0.03 |
|  |  |  |  | ENSDART00000078035-C | -16.60 |  | 0.04 |
| crybb2 | crystallin, beta B2 | NA | chr8:44670178-44676709 | ENSDART00000135525-C | -16.60 |  | 0.03 |
|  |  |  |  |  |  | -16.60 | <0.01 |
|  |  |  |  | ENSDART00000075553-C |  | -16.60 | <0.01 |
| crygm2e | crystallin, gamma M2e | NA | chr9:23113466-23114334 | ENSDART00000136014-C | -16.60 |  | 0.05 |
|  |  |  |  |  |  | -16.60 | <0.01 |
| crygm7 | crystallin, gamma M7 | NA | chr9:23026436-23027402 | ENSDART00000139189-NC | -16.60 |  | 0.04 |
|  |  |  |  |  |  | -16.60 | 0.05 |
|  |  |  |  | ENSDART00000059693-C |  | -16.60 | <0.01 |
| cstf3 | cleavage stimulation factor, 3' pre-RNA, subunit 3 | NA | chr18:44148408-44175987 | ENSDART00000052437-C | -16.60 |  | 0.02 |
|  |  |  |  | ENSDART00000025423-C |  | 20.70 | 0.02 |
| dbnla | drebrin-like a | NA | chr10:43723550-43737936 | ENSDART00000025691-C | 18.20 |  | 0.02 |
|  |  |  |  | ENSDART00000134632-NC |  | 19.90 | 0.04 |
| dnmt1 | DNA (cytosine-5-)-methyltransferase 1 | Cysteine and methionine metabolism | chr3:55442889-55458861 | ENSDART00000078973-C | 18.60 |  | 0.01 |
|  |  |  |  | ENSDART00000021977-C | -3.06 |  | 0.02 |
|  |  |  |  | ENSDART00000128041-C |  | 21.20 | 0.03 |
| eif2b4 | eukaryotic translation initiation factor 2B, subunit 4 delta | NA | chr20:19588813-19604704 | ENSDART00000026239-C | 19.50 |  | 0.03 |
|  |  |  |  | ENSDART00000125830-C |  | 14.20 | <0.01 |
| elavl1 | ELAV (embryonic lethal, abnormal vision, Drosophila)-like 1 (Hu antigen R) |  |  | ENSDART00000146123-C | -15.10 |  | <0.01 |
|  |  |  |  | ENSDART00000040427-C | 20.50 |  | 0.01 |
|  |  |  |  |  |  | 18.30 | 0.03 |
| ercc2 | excision repair cross-complementing rodent repair deficiency, complementation group 2 | Basal transcription factors;  Nucleotide excision repair | chr15:23033565-23045907 | ENSDART00000141618-C | -15.70 |  | <0.01 |
|  |  |  |  | ENSDART00000007925-C | 11.00 |  | 0.02 |
|  |  |  |  | ENSDART00000100683-C |  | 12.00 | 0.01 |
| espl1 | extra spindle poles like 1 | Cell cycle;  Oocyte meiosis | chr6:39599713-39631829 | ENSDART00000156645-C | 13.60 |  | 0.01 |
|  |  |  |  |  |  | 17.20 | <0.01 |
| esyt3 | extended synaptotagmin-like protein 3 | NA | chr9:24817196-24840377 | ENSDART00000027443-C | 11.70 |  | 0.01 |
|  |  |  |  | ENSDART00000129737-NC |  | -16.60 | 0.05 |
| fdx1 | ferredoxin 1 | NA | chr9:30554003-30578028 | ENSDART00000078904-C | -16.60 |  | 0.04 |
|  |  |  |  |  |  | -16.60 | 0.03 |
| fgfr1a | fibroblast growth factor receptor 1a | MAPK signaling pathway;  Adherens junction;  Regulation of actin cytoskeleton | chr8:53708708-53802579 | ENSDART00000127119-C | -13.00 |  | <0.01 |
|  |  |  |  | ENSDART00000074774-C |  | 11.80 | 0.03 |
|  |  |  |  | ENSDART00000147742-C |  | 17.90 | 0.03 |
| frya | furry homolog a (Drosophila) | NA | chr15:31043263-31156604 | ENSDART00000155902-C | 17.90 |  | 0.01 |
|  |  |  |  | ENSDART00000045921-C | -16.60 |  | 0.03 |
|  |  |  |  |  |  | -16.60 | 0.03 |
| glula | glutamate-ammonia ligase (glutamine synthase) a | Alanine, aspartate and glutamate metabolism;  Arginine and proline metabolism;  Nitrogen metabolism | chr2:19460033-19463977 | ENSDART00000146819-NC | -16.60 |  | 0.01 |
|  |  |  |  |  |  | -16.60 | <0.01 |
|  |  |  |  | ENSDART00000147472-C |  | 16.20 | 0.02 |
| GTF3A (2 of 3) | general transcription factor IIIA | NA | chr19:18002297-18005293 | ENSDART00000138678-C | -3.01 |  | 0.05 |
|  |  |  |  | ENSDART00000151194-C |  | 22.50 | 0.02 |
| herpud1 | homocysteine-inducible, endoplasmic reticulum stress-inducible, ubiquitin-like domain member 1 | NA | chr18:17259806-17267746 | ENSDART00000034287-C | 18.70 |  | 0.03 |
|  |  |  |  |  |  | 21.10 | 0.02 |
|  |  |  |  | ENSDART00000151850-C |  | 19.30 | 0.03 |
| hif1al | hypoxia-inducible factor 1, alpha subunit, like | NA | chr15:24834827-24858868 | ENSDART00000124696-C | -16.60 |  | 0.04 |
|  |  |  |  |  |  | -16.60 | 0.03 |
|  |  |  |  | ENSDART00000023421-C |  | 19.80 | 0.04 |
| hist2h3c | histone cluster 2, H3c | NA | chr25:36163120-36243649 | ENSDART00000154856-C | 18.10 |  | 0.01 |
|  |  |  |  |  |  | 21.20 | 0.04 |
| hnrnpub | heterogeneous nuclear ribonucleoprotein Ub | Spliceosome | chr20:35083241-35095243 | ENSDART00000122696-C | 19.30 |  | 0.04 |
|  |  |  |  |  |  | 21.30 | 0.03 |
| insb | preproinsulin b | Oocyte meiosis;  Regulation of autophagy;  mTOR signaling pathway;  Regulation of actin cytoskeleton;  Insulin signaling pathway;  Progesterone-mediated oocyte maturation | chr14:29583202-29588274 | ENSDART00000042850-C | -16.60 |  | <0.01 |
|  |  |  |  |  |  | -15.70 | <0.01 |
| irx4b | iroquois homeobox protein 4b | NA | chr19:28741740-28747641 | ENSDART00000133354-C | 18.20 |  | 0.01 |
|  |  |  |  | ENSDART00000052338-C |  | 17.10 | 0.02 |
| ist1 | increased sodium tolerance 1 homolog (yeast) | NA | chr7:57735830-57748061 | ENSDART00000135259-C | 20.00 |  | 0.02 |
|  |  |  |  |  |  | 20.90 | 0.02 |
|  |  |  |  | ENSDART00000137729-C | 19.50 |  | 0.03 |
| kmt2ba | NA | NA | chr19:9855074-9897818 | ENSDART00000136957-C | -16.60 |  | 0.03 |
|  |  |  |  |  |  | -12.40 | <0.01 |
|  |  |  |  | ENSDART00000091588-C | -16.60 |  | 0.03 |
| llgl1 | lethal giant larvae homolog 1 (Drosophila) | NA | chr3:39965136-40049546 | ENSDART00000102540-C | -15.20 |  | 0.02 |
|  |  |  |  |  |  | -7.82 | 0.03 |
| lrrfip1b | leucine rich repeat (in FLII) interacting protein 1b | NA | chr6:15426596-15503985 | ENSDART00000141597-C | 19.40 |  | <0.01 |
|  |  |  |  | ENSDART00000143502-C |  | 17.90 | <0.01 |
| macf1 | microtubule-actin crosslinking factor 1 | NA | chr19:36588200-36899421 | ENSDART00000146394-C | 18.00 |  | 0.01 |
|  |  |  |  | ENSDART00000054274-C |  | -9.95 | 0.02 |
| map3k5 | mitogen-activated protein kinase kinase kinase 5 | MAPK signaling pathway | chr20:3038769-3120460 | ENSDART00000046641-C | 9.54 |  | 0.02 |
|  |  |  |  |  |  | 8.42 | 0.03 |
|  |  |  |  | ENSDART00000152495-C | -16.60 |  | 0.03 |
| march7 | NA | NA | chr6:11671695-11723171 | ENSDART00000151717-C | 17.30 |  | 0.03 |
|  |  |  |  | ENSDART00000150989-C |  | 19.10 | 0.05 |
| mark2a | MAP/microtubule affinity-regulating kinase 2a | NA | chr21:26598188-26668126 | ENSDART00000143239-C | 19.50 |  | 0.01 |
|  |  |  |  | ENSDART00000040754-C |  | 19.00 | 0.02 |
| mbnl3 | muscleblind-like 3 (Drosophila) | NA | chr14:31859432-31929544 | ENSDART00000113479-C | 18.40 |  | 0.03 |
|  |  |  |  | ENSDART00000111691-C | -16.60 |  | 0.03 |
|  |  |  |  |  |  | -16.60 | 0.04 |
| mcamb | melanoma cell adhesion molecule b | NA | chr15:22625409-22696300 | ENSDART00000020425-C | -16.60 |  | 0.04 |
|  |  |  |  | ENSDART00000115362-C |  | -16.60 | 0.04 |
| Metazoa_SRP | Metazoan signal recognition particle RNA | NA | chr11:12243966-12244249 | ENSDART00000126039-NC | -16.60 |  | <0.01 |
|  |  |  |  |  |  | -16.60 | <0.01 |
|  |  |  |  | ENSDART00000130848-NC | -2.61 |  | 0.01 |
|  |  |  |  | ENSDART00000129039-NC | -2.39 |  | 0.03 |
|  |  |  |  | ENSDART00000122404-NC | -2.08 |  | 0.03 |
|  |  |  |  | ENSDART00000128327-NC | -2.14 |  | 0.03 |
|  |  |  |  | ENSDART00000126698-NC | -2.44 |  | 0.03 |
|  |  |  |  | ENSDART00000121803-NC | -2.26 |  | 0.04 |
|  |  |  |  | ENSDART00000129137-NC | -1.98 |  | 0.04 |
|  |  |  |  | ENSDART00000123473-NC | -2.09 |  | 0.04 |
|  |  |  |  |  |  | -2.88 | 0.01 |
|  |  |  |  | ENSDART00000123279-NC |  | -2.44 | 0.01 |
|  |  |  |  | ENSDART00000126933-NC |  | -2.41 | 0.03 |
|  |  |  |  | ENSDART00000125239-NC |  | 1.55 | 0.04 |
|  |  |  |  | ENSDART00000126476-NC |  | 20.70 | 0.05 |
| mkln1 | muskelin 1, intracellular mediator containing kelch motifs | NA | chr4:10674929-10795292 | ENSDART00000137736-C | 14.00 |  | 0.01 |
|  |  |  |  |  |  | 19.30 | 0.04 |
|  |  |  |  | ENSDART00000150275-NC |  | -16.60 | 0.04 |
| mpx | myeloid-specific peroxidase | NA | chr10:6807369-6814452 | ENSDART00000111058-C | 18.90 |  | 0.04 |
|  |  |  |  | ENSDART00000043961-C |  | 17.90 | 0.04 |
| nckap1 | NCK-associated protein 1 | Regulation of actin cytoskeleton | chr9:45789618-45893194 | ENSDART00000132696-C | 20.30 |  | 0.05 |
|  |  |  |  |  |  | 19.60 | 0.04 |
|  |  |  |  | ENSDART00000086176-C |  | 17.50 | 0.04 |
| ncoa6 | nuclear receptor coactivator 6 | NA | chr23:2714971-2737144 | ENSDART00000147953-C | -16.60 |  | 0.04 |
|  |  |  |  | ENSDART00000105284-C |  | -12.80 | 0.01 |
| ndrg4 | N-myc downstream regulated gene 4 | NA | chr25:12560692-12618755 | ENSDART00000151830-C | -16.60 |  | 0.02 |
|  |  |  |  |  |  | -16.60 | 0.02 |
|  |  |  |  | ENSDART00000089737-C |  | 19.30 | 0.01 |
|  |  |  |  | ENSDART00000128808-C |  | -16.60 | 0.02 |
| ndufs2 | NADH dehydrogenase (ubiquinone) Fe-S protein 2 | Oxidative phosphorylation | chr7:20254851-20279641 | ENSDART00000079762-C | -10.80 |  | 0.01 |
|  |  |  |  | ENSDART00000108787-C | 21.50 |  | 0.04 |
|  |  |  |  |  |  | 19.90 | 0.04 |
| nipblb | nipped-b homolog b (Drosophila) | NA | chr10:37719939-37791209 | ENSDART00000108484-C | -16.60 |  | 0.04 |
|  |  |  |  |  |  | -13.90 | <0.01 |
| nop56 | NOP56 ribonucleoprotein homolog | NA | chr21:10382529-10405532 | ENSDART00000102433-C | -16.60 |  | 0.01 |
|  |  |  |  | ENSDART00000135696-NC |  | 20.20 | 0.05 |
| nr1i2 | nuclear receptor subfamily 1, group I, member 2 | NA | chr9:9724021-9786561 | ENSDART00000125174-C | 15.10 |  | <0.01 |
|  |  |  |  | ENSDART00000123247-C |  | -16.60 | 0.04 |
| nup205 | nucleoporin 205 | NA | chr18:11684501-11719423 | ENSDART00000143923-C | -16.60 |  | 0.03 |
|  |  |  |  |  |  | -16.60 | 0.03 |
| ogt.1 | O-linked N-acetylglucosamine (GlcNAc) transferase, tandem duplicate 1 | O-Mannosyl glycan biosynthesis | chr14:18587709-18646065 | ENSDART00000080496-C | 19.10 |  | 0.03 |
|  |  |  |  |  |  | 20.60 | 0.01 |
|  |  |  |  | ENSDART00000122502-C |  | -14.00 | <0.01 |
| pik3r3a | phosphoinositide-3-kinase, regulatory subunit 3a (gamma) | ErbB signaling pathway;  Phosphatidylinositol signaling system;  mTOR signaling pathway;  Apoptosis;  VEGF signaling pathway;  Focal adhesion;  Toll-like receptor signaling pathway;  Jak-STAT signaling pathway;  Natural killer cell mediated cytotoxicity;  Regulation of actin cytoskeleton;  Insulin signaling pathway;  Progesterone-mediated oocyte maturation | chr2:3506204-3567452 | ENSDART00000126431-C | 19.30 |  | 0.04 |
|  |  |  |  | ENSDART00000157017-C |  | 13.20 | <0.01 |
| plk3 | polo-like kinase 3 (Drosophila) | NA | chr2:35324469-35332623 | ENSDART00000133018-NC | 20.30 |  | 0.03 |
|  |  |  |  |  |  | 20.80 | 0.04 |
| polk | polymerase (DNA directed) kappa | NA | chr5:47506860-47525564 | ENSDART00000141039-C | -16.60 |  | 0.05 |
|  |  |  |  |  |  | -16.90 | <0.01 |
| ppp1r14ba | protein phosphatase 1, regulatory (inhibitor) subunit 14Ba | NA | chr21:26390669-26405231 | ENSDART00000144342-C | 19.30 |  | 0.01 |
|  |  |  |  |  |  | 18.30 | 0.03 |
|  |  |  |  | ENSDART00000065398-C | 18.70 |  | 0.04 |
| ptgdsa | prostaglandin D2 synthase a | NA | chr10:10754706-10762832 | ENSDART00000139143-C | -16.60 |  | <0.01 |
|  |  |  |  | ENSDART00000101077-C |  | 19.60 | 0.03 |
| rab11bb | RAB11B, member RAS oncogene family, b | NA | chr2:56673948-56688176 | ENSDART00000036452-C | 18.60 |  | 0.02 |
|  |  |  |  | ENSDART00000123392-C |  | 17.50 | 0.04 |
| ralgapa1 | Ral GTPase activating protein, alpha subunit 1 (catalytic) | NA | chr17:12630934-12787578 | ENSDART00000154984-C | -16.60 |  | 0.05 |
|  |  |  |  |  |  | -16.60 | 0.05 |
| rbm39a | RNA binding motif protein 39a | NA | chr11:25272889-25281203 | ENSDART00000135435-C | -16.60 |  | 0.02 |
|  |  |  |  |  |  | -16.60 | 0.02 |
|  |  |  |  | ENSDART00000139298-NC |  | 19.90 | 0.03 |
| retsatl | retinol saturase (all-trans-retinol 13,14-reductase) like | Retinol metabolism | chr9:46061596-46066105 | ENSDART00000043899-C | 20.40 |  | 0.01 |
|  |  |  |  |  |  | 21.70 | 0.02 |
|  |  |  |  | ENSDART00000141434-C | -2.38 |  | 0.03 |
| ripk1l | receptor (TNFRSF)-interacting serine-threonine kinase 1, like | Apoptosis;  Toll-like receptor signaling pathway;  RIG-I-like receptor signaling pathway;  Cytosolic DNA-sensing pathway | chr2:1088132-1104351 | ENSDART00000024967-C | 18.10 |  | 0.01 |
|  |  |  |  | ENSDART00000109397-C |  | 16.80 | 0.03 |
| rps18 | ribosomal protein S18 | Ribosome | chr19:18309519-18318937 | ENSDART00000142769-C | -16.60 |  | 0.05 |
|  |  |  |  | ENSDART00000131839-C |  | 5.38 | 0.05 |
| rps2 | ribosomal protein S2 | Ribosome | chr3:18127815-18132119 | ENSDART00000138107-C | 23.10 |  | 0.05 |
|  |  |  |  |  |  | 23.10 | 0.01 |
| sdr39u1 | short chain dehydrogenase/reductase family 39U, member 1 | NA | chr2:37536501-37585260 | ENSDART00000034512-C | 17.70 |  | 0.02 |
|  |  |  |  | ENSDART00000150810-C |  | 2.60 | 0.03 |
| si:ch211-119d14.4 | si:ch211-119d14.4 | NA | chr15:24360756-24363948 | ENSDART00000155236-C | 16.70 |  | 0.04 |
|  |  |  |  | ENSDART00000154409-C |  | 16.40 | 0.04 |
| si:ch73-204p21.2 | si:ch73-204p21.2 | NA | chr17:15887243-15925593 | ENSDART00000154554-C | 10.20 |  | 0.04 |
|  |  |  |  | ENSDART00000155005-C |  | -6.69 | 0.05 |
| si:dkey-102m7.3 | si:dkey-102m7.3 | NA | chr14:18736451-18738525 | ENSDART00000140355-NC | -16.60 |  | 0.04 |
|  |  |  |  | ENSDART00000132532-NC |  | 15.90 | 0.05 |
| si:dkey-196h17.4 | si:dkey-196h17.4 | NA | chr13:6709813-6716236 | ENSDART00000145073-C | 18.80 |  | 0.01 |
|  |  |  |  | ENSDART00000145759-C |  | -16.60 | <0.01 |
| si:dkey-262k9.2 | si:dkey-262k9.2 | NA | chr15:35664041-35713646 | ENSDART00000155100-C | -14.10 |  | 0.01 |
|  |  |  |  | ENSDART00000154552-C | 15.80 |  | 0.03 |
|  |  |  |  |  |  | 15.80 | 0.01 |
| slc12a8 | solute carrier family 12 (potassium/chloride transporters), member 8 | NA | chr9:39697284-39734546 | ENSDART00000110651-C | 17.00 |  | 0.05 |
|  |  |  |  | ENSDART00000135774-C |  | 17.30 | 0.03 |
| slc38a4 | solute carrier family 38, member 4 | NA | chr4:1375770-1425627 | ENSDART00000067446-C | -16.00 |  | <0.01 |
|  |  |  |  | ENSDART00000150198-C |  | -16.60 | 0.05 |
| sltm | SAFB-like, transcription modulator | NA | chr7:31873722-31896958 | ENSDART00000142818-C | 19.30 |  | 0.04 |
|  |  |  |  |  |  | 18.10 | 0.04 |
|  |  |  |  | ENSDART00000139546-C |  | -16.60 | 0.04 |
|  |  |  |  | ENSDART00000138298-NC |  | -16.60 | 0.04 |
| smarcc1b | SWI/SNF related, matrix associated, actin dependent regulator of chromatin, subfamily c, member 1b | NA | chr19:18923988-18960933 | ENSDART00000104385-C | -8.32 |  | 0.03 |
|  |  |  |  | ENSDART00000085619-C |  | -16.60 | 0.03 |
| sulf2b | sulfatase 2b | NA | chr23:15314865-15441274 | ENSDART00000104904-C | 18.10 |  | 0.05 |
|  |  |  |  | ENSDART00000035865-C |  | 17.20 | 0.01 |
| syt10 | synaptotagmin X | NA | chr4:9953285-9973630 | ENSDART00000067258-C | -16.60 |  | <0.01 |
|  |  |  |  | ENSDART00000136049-C |  | 13.70 | 0.01 |
| taf1b | TATA box binding protein (Tbp)-associated factor, RNA polymerase I, B | NA | chr20:29539377-29558918 | ENSDART00000049224-C | -16.60 |  | <0.01 |
|  |  |  |  | ENSDART00000062377-C | 11.60 |  | 0.01 |
|  |  |  |  |  |  | 12.80 | 0.01 |
| tenm3 | teneurin transmembrane protein 3 | NA | chr1:39750141-40142496 | ENSDART00000137676-C | 17.90 |  | 0.04 |
|  |  |  |  | ENSDART00000084891-C | -16.60 |  | 0.05 |
|  |  |  |  |  |  | -16.60 | 0.04 |
| tjp2b | tight junction protein 2b (zona occludens 2) | NA | chr8:11975936-12067805 | ENSDART00000115057-C | -8.87 |  | 0.03 |
|  |  |  |  | ENSDART00000018739-C | -16.60 |  | 0.03 |
|  |  |  |  |  |  | -16.60 | 0.03 |
|  |  |  |  | ENSDART00000092355-C |  | 20.10 | 0.02 |
| tnnt3b | troponin T3b, skeletal, fast | NA | chr7:40837568-40853085 | ENSDART00000141049-NC | 19.10 |  | 0.02 |
|  |  |  |  |  |  | 17.60 | 0.04 |
| tspan3a | tetraspanin 3a | NA | chr18:26843682-27032377 | ENSDART00000130854-C | 21.50 |  | 0.02 |
|  |  |  |  | ENSDART00000127783-C |  | 21.30 | 0.02 |
| U1 | U1 spliceosomal RNA | NA | chr22:37466962-37475623 | ENSDART00000117980-NC | 23.90 |  | 0.03 |
|  |  |  |  | ENSDART00000131186-NC |  | 30.70 | 0.03 |
| U3 | Small nucleolar RNA U3 | NA | chr14:11830992-11831207 | ENSDART00000129406-NC | -16.60 |  | <0.01 |
|  |  |  |  | ENSDART00000124796-NC | -16.60 |  | <0.01 |
|  |  |  |  | ENSDART00000122828-NC | -16.60 |  | <0.01 |
|  |  |  |  | ENSDART00000129051-NC | -16.60 |  | <0.01 |
|  |  |  |  | ENSDART00000127439-NC | -16.60 |  | <0.01 |
|  |  |  |  | ENSDART00000124006-NC | -16.60 |  | <0.01 |
|  |  |  |  | ENSDART00000121956-NC | -3.91 |  | 0.01 |
|  |  |  |  | ENSDART00000130213-NC | -7.66 |  | 0.01 |
|  |  |  |  | ENSDART00000118424-NC | -2.71 |  | 0.02 |
|  |  |  |  |  |  | -2.44 | 0.01 |
|  |  |  |  | ENSDART00000121471-NC | 23.80 |  | 0.02 |
|  |  |  |  |  |  | 25.20 | <0.01 |
|  |  |  |  | ENSDART00000128221-NC | -16.60 |  | 0.03 |
|  |  |  |  | ENSDART00000127574-NC | -3.26 |  | 0.03 |
|  |  |  |  | ENSDART00000127880-NC | -5.24 |  | 0.05 |
|  |  |  |  | ENSDART00000127373-NC |  | 27.40 | <0.01 |
|  |  |  |  | ENSDART00000125076-NC |  | 23.60 | 0.03 |
| ubr5 | ubiquitin protein ligase E3 component n-recognin 5 | Ubiquitin mediated proteolysis | chr16:56147790-56252683 | ENSDART00000134444-C | 16.30 |  | <0.01 |
|  |  |  |  | ENSDART00000128437-C | -16.60 |  | 0.05 |
|  |  |  |  |  |  | -16.60 | 0.03 |
| wee2 | WEE1 homolog 2 (S. pombe) | Cell cycle | chr18:20341152-20346693 | ENSDART00000151974-C | -14.50 |  | <0.01 |
|  |  |  |  | ENSDART00000136710-C |  | -16.60 | 0.02 |
| zfyve21 | zinc finger, FYVE domain containing 21 | NA | chr13:44268926-44288601 | ENSDART00000042588-C | 12.50 |  | <0.01 |
|  |  |  |  | ENSDART00000074728-C | -16.60 |  | 0.04 |
|  |  |  |  |  |  | -16.60 | 0.04 |
| zgc:165555 | zgc:165555 | NA | chr25:36163120-36243649 | ENSDART00000130395-C | 17.70 |  | 0.01 |
|  |  |  |  |  |  | 19.90 | 0.01 |
|  |  |  |  | ENSDART00000126086-C |  | 24.80 | <0.01 |
| zgc:165582 | zgc:165582 | NA | chr3:52730620-52748831 | ENSDART00000104650-C | 17.90 |  | 0.01 |
|  |  |  |  | ENSDART00000155334-C |  | 16.40 | 0.04 |
| zgc:171759 | zgc:171759 | NA | chr25:37347967-37386645 | ENSDART00000146977-C | 17.70 |  | 0.01 |
|  |  |  |  | ENSDART00000128017-C | 19.50 |  | 0.03 |
|  |  |  |  | ENSDART00000155850-C | 23.60 |  | 0.03 |
|  |  |  |  |  |  | 27.00 | 0.01 |
|  |  |  |  | ENSDART00000152735-C | -16.60 |  | 0.04 |
|  |  |  |  | ENSDART00000146289-C | -16.60 |  | 0.04 |
| zgc:175136 | zgc:175136 | NA | chr19:30560526-30564146 | ENSDART00000132237-C | -2.36 |  | 0.02 |
|  |  |  |  | ENSDART00000131999-NC |  | -16.60 | 0.05 |
| zgc:64002 | zgc:64002 | NA | chr11:12487640-12497046 | ENSDART00000029595-C | -16.60 |  | 0.02 |
|  |  |  |  |  |  | -16.60 | 0.02 |
|  |  |  |  | ENSDART00000125060-C | 17.80 |  | 0.04 |
| zgc:92533 | zgc:92533 | NA | chr19:5980623-6004973 | ENSDART00000151644-NC | 19.30 |  | 0.04 |
|  |  |  |  | ENSDART00000146890-C |  | -1.27 | 0.02 |
| zp3b | zona pellucida glycoprotein 3b | NA | chr2:5895635-5901186 | ENSDART00000058256-C | -15.50 |  | 0.01 |
|  |  |  |  | ENSDART00000139429-C |  | 22.50 | 0.01 |
|  |  |  |  | ENSDART00000153328-NC |  | -12.50 | 0.01 |
| zranb3 | zinc finger, RAN-binding domain containing 3 | NA | chr22:12387932-12414329 | ENSDART00000136408-C | 18.30 |  | 0.05 |
|  |  |  |  | ENSDART00000123231-C |  | 19.20 | 0.02 |

**Note:** (1) In the fifth column of Table 1, “C” following each transcript indicates that this transcript can be translated into protein; (2) “NC” following each transcript indicates that this transcript can not be translated into protein.
