# Supplementary material for: Immunotoxicity of β-Diketone Antibiotic Mixtures to Zebrafish (Danio rerio) by Transcriptome Analysis
Source: PLoS One. 2016 Apr 5;11(4):e0152530. doi: 10.1371/journal.pone.0152530 (PMC4821563; doi:10.1371/journal.pone.0152530)
Supplement: S4 Table — (DOC) [file pone.0152530.s007.doc]

**S4 Table.** The mostly enriched KEGG pathway of the differentially expressed genes

| Pathway id. | Pathway name | Gene number annotated in KEGG pathway | Background  gene number | *p*-value |
| --- | --- | --- | --- | --- |
| ko03040 | Spliceosome | 2 | 130 | <0.01 |
| ko04110 | Cell cycle | 2 | 133 | <0.01 |
| ko04120 | Ubiquitin mediated proteolysis | 1 | 138 | <0.01 |
| ko04510 | Focal adhesion | 1 | 230 | <0.01 |
| ko04145 | Phagosome | 1 | 157 | <0.01 |
| ko04010 | MAPK signaling pathway | 2 | 308 | <0.01 |
| ko04142 | Lysosome | 1 | 142 | <0.01 |
| ko04910 | Insulin signaling pathway | 2 | 166 | <0.01 |
| ko04114 | Oocyte meiosis | 2 | 144 | <0.01 |
| ko00190 | Oxidative phosphorylation | 2 | 160 | <0.01 |
| ko04810 | Regulation of actin cytoskeleton | 5 | 261 | <0.01 |
| ko04520 | Adherens junction | 1 | 98 | <0.01 |
| ko03420 | Nucleotide excision repair | 1 | 45 | <0.01 |
| ko04210 | Apoptosis | 2 | 93 | <0.01 |
| ko04622 | RIG-I-like receptor signaling pathway | 1 | 56 | <0.01 |
| ko03022 | Basal transcription factors | 1 | 45 | <0.01 |
| ko04914 | Progesterone-mediated oocyte maturation | 2 | 109 | <0.01 |
| ko04012 | ErbB signaling pathway | 1 | 106 | <0.01 |
| ko04650 | Natural killer cell mediated cytotoxicity | 1 | 99 | <0.01 |
| ko04620 | Toll-like receptor signaling pathway | 2 | 98 | <0.01 |
| ko04370 | VEGF signaling pathway | 1 | 96 | <0.01 |
| ko04630 | Jak-STAT signaling pathway | 1 | 112 | <0.01 |
| ko04623 | Cytosolic DNA-sensing pathway | 1 | 30 | <0.01 |
| ko00514 | O-Mannosyl glycan biosynthesis | 1 | 38 | <0.01 |
| ko00240 | Pyrimidine metabolism | 1 | 161 | <0.01 |

**Note: (1)** The data in the 3^th^ column indicate the gene number among the 106 genes, which can be annotated in KEGG pathway; (2) If A and B indicate the ratios of genes, with significant difference, to those of total genes in each and all KEGG pathway, respectively, the *p*-value shows whether there is significant difference between A and B.
